# Acute effect of a Mediterranean-style dietary pattern (MDP) on mood, anxiety and cognition in UK adults with mild to moderate anxiety and depression: the MediMood randomised controlled trial protocol

**DOI:** 10.1136/bmjopen-2023-082935

**Published:** 2024-12-20

**Authors:** Latife Esgunoglu, Marrium Liaquat, Rachel Gillings, Alpar Lazar, Adrian Leddy, Jon Brooks, William Penny, Saber Sami, M Hornberger, Emma Stevenson, Amy Jennings, Anne Marie Minihane

**Affiliations:** 1 Norwich Medical School, University of East Anglia, Norwich, UK; 2 School of Health Sciences, University of East Anglia, Norwich, UK; 3 School of Psychology, University of East Anglia, Norwich, UK; 4 Institute of Cellular Medicine, Newcastle University, Newcastle upon Tyne, UK; 5 Norwich Institute for Healthy Aging, Norwich, UK

**Keywords:** NUTRITION & DIETETICS, MENTAL HEALTH, Clinical Trial

## Abstract

**Introduction:**

Psychological disorders including depression and anxiety are significant public health concerns. A Mediterranean-style dietary pattern (MDP) has been associated with improved mental well-being in observational studies. Evidence of the acute (defined as postprandial to 1 week) effects of an MDP on brain function, mood, cognition and important modulators, including sleep and the gut microbiota is limited. The current intervention aims to examine whether an MDP, compared with a Western diet (WD), improves mood, cognition and anxiety symptoms, postprandially, at 24-hour and after 5 days in adults with mild to moderate anxiety and depression.

**Methods and analysis:**

Twenty-five UK adults (aged 18 or over) with mild to moderate anxiety and/or depression and low adherence to an MDP were recruited to a cross-over randomised controlled trial. Each participant undergoes a 5 day MDP and a 5 day WD in a randomised order with all meals provided. The co-primary outcomes are mood and anxiety, with secondary outcomes including cognitive function, brain perfusion (as assessed by MRI), sleep quality, blood pressure, plasma glucose, insulin, lipids, C-reactive protein, cortisol, brain-derived neurotrophic factor, gut microbiota speciation and microbial metabolites including short chain fatty acids. A linear mixed model and/or paired analysis will be used to compare the effects of treatments over time.

**Ethics and dissemination:**

The study has received a favourable ethics opinion from the National Health Service London Queen Square Research Ethics Committee (22/LO/0796). The results will be disseminated through scientific journals and conferences.

**Trial registration number:**

NCT05927376.

**STRENGTHS AND LIMITATIONS OF THIS STUDY**

- The design of MediMood was informed by a systematic review of the literature which provided the need for, and informed the design of, the current randomised controlled trial (RCT).
- MediMood is a highly controlled efficacy RCT with all food provided for 5 days with detailed food preparation instructions rather than dietary advice only.
- The study quantified key physiological determinants of brain health including gut microbiota and brain perfusion quantified by MRI.
- To minimise participant burden, the primary outcomes (mood and anxiety) and biological samples collection could not be conducted daily.
- For logistical and costs reasons, MRI scans could not be executed at the beginning and at the end of the 5 day interventions, to assess short-term changes in brain perfusion, limiting us to assessing only the postprandial effects of dietary intervention on cerebral blood flow.

## Introduction

 Mental health disorders represent a major public health challenge.^1^ In 2019, depression exceeded 280 million cases globally, and anxiety surpassed 300 million cases, as the two most common forms of mental health disorders.^1^ Mental health disorders have constituted around 15% of ‘years lived with disability’ worldwide since 1990,^2^ with depression predicted to be the global leading cause of disease by 2030.^3^ In England, nearly 20% of adults report depression, anxiety, sleep problems, poor concentration and forgetfulness.^4^

The economic impact of mental health disorders are substantial, with an estimated annual global cost of approximately $5 trillion including loss of productivity.^5^ The UK National Health Services (NHS) has allocated a £2.3 billion budget for the years 2023–2024 for mental health services as part of its long-term plan.^6^

The main treatment for mental health disorders are antidepressant medications and psychotherapy; both can cause negative side effects,^7^ stigma^8^ and have poor uptake.^4^ Despite increased treatment in recent decades, no decrease in the prevalence of mental disorders is evident,^9^ underlining the need for alternative intervention approaches.

The WHO has highlighted the critical need for ‘affordable, effective and feasible strategies to promote, protect and restore mental health’, and launched several initiatives such as the ‘Comprehensive Mental Health Action Plan 2013–2030’^10^ and the ‘World mental health report: transforming mental health for all’^11^ to address these needs.

A Mediterranean-style dietary pattern (MDP) consists of high amounts of fruits, vegetables, legumes, nuts, olive oil and fish. It is low in high fat dairy, red and processed meat, carbonated beverages and free sugars, and rich in unsaturated fatty acids, polyphenols and unrefined complex carbohydrates,^12^ which aligns with healthy eating guidelines in the UK and many other countries.^13^

Long-term adherence to an MDP has been consistently shown to protect mental health. Longitudinal analysis of the SUN cohort (n=10 094) reported that higher MDP adherence was correlated with a lower depression incidence after 4.4 years,^14^ supported by a meta-analysis of observational studies showing a reduced risk of depression associated with long-term MDP adherence (OR=0.72; 95% CI, 0.60 to 0.87).^15^ The Prevencion con Dieta Mediterranea (PREDIMED), the hallmark randomised controlled trial (RCT) in the field, reported a 41% reduction in depression among at-risk individuals with type 2 diabetes who followed an MDP supplemented with nuts for 3 years (HR=0.59; 95% CI, 0.36 to 0.98).^16^ The HELFIMED,^17^ SMILES^18^ and AMMEND^19^ trials, all of which examined the effects of an MDP on depression in adults with moderate to severe depression over the course of 3–6 months, demonstrated significant decrease in depressive symptoms. The cognitive benefits of an MDP have also been consistently reported. The PREDIMED study showed improved cognition after MDP interventions,^20^ while a recent UK Biobank analysis suggested a reduced risk of future dementia associated with MDP consumption.^21^ Additionally, a meta-analysis reported a linear dose–response relationship between an MDP adherence and the risk of future cognitive disorders.^22^

On the other hand, a Western diet (WD), which includes high amounts of saturated fat (SFA) and simple sugars, is associated with compromised brain health, and a higher incidence of depression, anxiety and neurological conditions.^23 24^

Our systematic review investigating the short-term effects (up to 10 days) of an MDP on brain health revealed improved mood and cognition, in particular, alertness, contentment and attention domains in the four included studies.^25^ There were too few studies to draw firm conclusions, and we identified several limitations and research gaps. Three of the four studies were of 10 days duration, with no shorter term or postprandial data available. Besides, in all reviewed studies, participants were provided with dietary advice rather than the intervention diet, and adherence to the intervention was not monitored. Furthermore, mental health outcomes were not comprehensively assessed to elucidate which domains are most responsive to a short-term MDP intervention and little attention has been given to possible underlying mechanisms which could be mediating the acute effects of an MDP such as changes in inflammation, glucose regulation, cerebral blood flow (CBF) and the gut microbiota.^25^

Therefore, despite its potential benefits^25^ on mental well-being and quality of life, the acute effects of an MDP are largely unknown. The overall aim of MediMood study is to examine the impact of an MDP versus a Western-style diet (WD) on mood, anxiety and cognition postprandially, at 24-hour (mood and anxiety only) and after 5 days, and to investigate underpinning physiological mechanisms.

## Methods and analysis

This article follows the Standard Protocol Items: Recommendations for Interventional Trials (SPIRIT) guidelines.^26^

### Study setting

MediMood is a single-centre cross-over RCT conducted at the University of East Anglia (UEA), and the NHS Clinical Research Facility (CRF) intervention centre, based at the Quadram Institute (QI), in Norwich, UK. The data collection period spanned from June to December 2023.

### Eligibility criteria

Potential participants were recruited from the general population and from the University staff and students, using advertising posters/leaflets, internal emails and social media.

Twenty-five people aged 18 years or over were recruited. Participants were eligible if they met the following conditions:

1. Had mild to moderate level depression and/or anxiety, established using the Patient Health Questionnaire (PHQ-9) (score 5-14/27)^27^ and the Generalised Anxiety Disorder (GAD-7) (score 5-14/21).^28^ Both measures are commonly used in the NHS settings as preindicators of depression and anxiety.
2. Were not already following an MDP, established using the Mediterranean Diet Adherence Screener (MEDAS) (score≤7/14) (online supplemental appendix 1).^29^
3. Had been on the same dosage of their medication for at least 3 months and expected to keep a stable dosage for the next 3 months (for those who are on any antidepressant/antianxiety medication).
4. Were eligible to undertake an MRI scan (eg, not having any possibility of pregnancy).
5. Were not vegan or vegetarian.
6. Did not have food allergies or intolerances to the food provided such as fish and nuts.

If participants reported antibiotics use in the last month, their participation was postponed until 1 month after treatment to allow the gut microbiota composition to return to its habitual status. Participants are requested to keep any probiotic supplement use and physical activity levels stable during their participation. For MRI safety, ‘any possibility of being pregnant’ or those with specific medical implants or devices (such as cardiac pacemakers or artificial limbs) were precluded from participating (online supplemental appendix 2, MRI Safety Screening Form). Participants were advised to discuss their participation with their general practitioners (GPs) and informed that the study researchers were going to inform their GPs about their participation (online supplemental appendix 3, Participant Information Sheet).

Table 1 lists the full inclusion and exclusion criteria.

**Table 1:** The eligibility criteria

Inclusion criteria	Exclusion criteria
Males and females aged 18 or over	Vegan, vegetarian
Mild to moderate level anxiety and/or depression (PHQ-9 and/or GAD-7 scores of 5–14)	Allergic to any of the study components for example, nuts and fish
Low MDP adherence (MEDAS score≤7/14)	On antianxiety and/or antidepressant medication which has changed in the last 3 months or likely to change in the next 3 months
Able to have an MRI scan	Unwilling or unable to make changes to their diet for 10 days (2×5 days period)
Computer literate with internet access	Unable to attend the intervention centre
Fluent in written and spoken English	MEDAS score>7
Gave consent for the study team to contact their GP	Not fluent in written and spoken English
Willing and able to comply with all study procedures including diet	MRI unsafety
	Not agreement for the study team to contact their GP
	Not prepared to make changes to diet for 10 days (2×5 days period)

GAD-7Generalised Anxiety Disorder (includes 7-item)GPgeneral practitionerMDPMediterranean-style dietary patternMEDASMediterranean Diet Adherence Screener tool (includes 14-items)PHQ-9Patient Health Questionnaire (includes 9-item)

### Recruitment

Individuals who expressed an interest in the study were provided with the Participant Information Sheet and directed to the study website (https://app.mantal.co.uk/medimood), built on the Mantal platform, an online research management portal. First, participants were asked to provide consent (online supplemental appendix 4, Consent Form). Second, participants completed questionnaires to ascertain if they meet the study inclusion criteria detailed above. Those meeting the criteria were enrolled in the study and randomised to either an MDP or a WD for arm 1 of the study, by using random number generator in Microsoft Excel.

**Figure 1:**
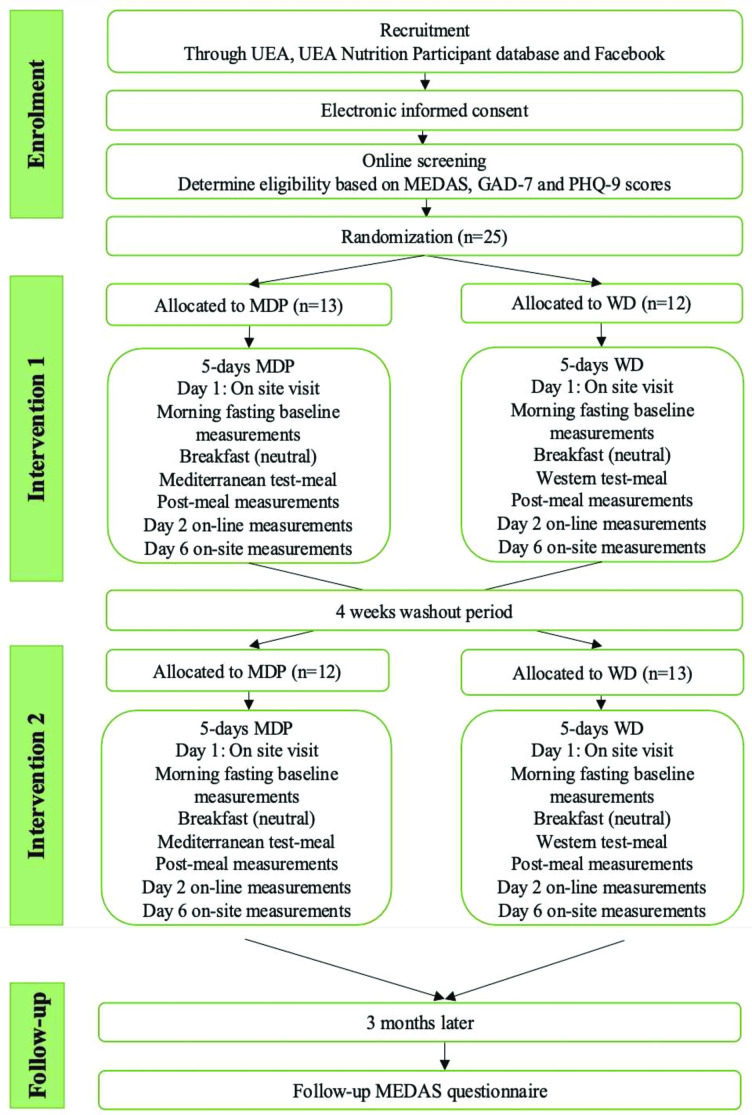
Study flow diagram. GAD-7, Generalised Anxiety Disorder (includes 7-item); MEDAS, Mediterranean Diet Adherence Screener; PHQ-9, Patient Health Questionnaire (includes 9-item); WD, Western-style diet.

The study stages are displayed in figure 1.

### Safeguards for maintaining psychological well-being of participants

Enrolled participants’ GPs are notified about their patients’ participation and provided with their PHQ-9 and GAD-7 scores. The GPs of participants who are ineligible due to severe levels of anxiety and/or depression were also notified. All participants are signposted to mental health and well-being support.

### Interventions

The experimental arm is a 5 day MDP, with a 5 day WD comparator arm. Both diets are designed to provide approximately 2000 kcal/day (±10% flexibility per day, ranging between 1800 and 2200 kcal). The MDP diet scores 14 (or 13 if no alcohol is consumed) on the MEDAS scale (maximum score 14) on each of the 5 days. Conversely, the WD scores zero points on the MEDAS scale on each of the 5 days. The full meal plans are presented in the online supplemental appendix 5.

The total macronutrient (carbohydrates, fat and protein) composition, and free sugars, fibre, SFA and monounsaturated fat content of the diets have been designed to ensure that they represent typical MDP and WD. For the MDP, the PREDIMED diet was used as the reference standard^30^ and for the WD, the nutrient profile was based on extreme nutrient intakes (lowest or highest 2.5%) of the UK population using the UK National Diet and Nutrition Survey (NDNS) data (https://www.gov.uk/government/collections/national-diet-and-nutrition). Table 2 represents the nutrient compositions of the test lunch meals, and table 3 represents the nutrient compositions of the full 5 day diets.

**Table 2:** Nutrient composition of the lunch test meals (day 1) taken from the product labels

		Mediterranean diet	Western diet
Energy	Kcal	1013	984
Carbohydrates	g	45	123
	%	18.3	50.3
Free sugars	g	0	83
Fibre	g	10.6	2.9
Proteins	g	60	37.5
	%	24.3	15.3
Total fat	g	63	37.3
	%	57.4	34.4
SFA	g	8.8	15.2
	%	7.8	14

%, contribution to the total daily energy intake as per cent; g, grams; kcal, kilocalories; SFA, saturated fatty acids

**Table 3:** Nutrient composition table of the 5 day test diets

		Mediterranean diet (mean±SD)	Western diet (mean±SD)
Energy	Kcal/day	1878±46	2027±79
Carbohydrates	g/day	154.2±16.2	230.8±24
	%	32.8±3.0	45.5±4.6
Free sugars	g/day	0.3±0.6	35.7±10.4
Fibre	g/day	34.8±6.4	10.6±2.9
Proteins	g/day	80.8±19.1	64.6±11.4
	%	17.1±4	12.7±2.3
Total fat	g/day	105.0±8.9	94.2±11.2
	%	50.3±4.6	41.8±4.6
SFA	g/day	16.1±2.0	37.1±3.9
	%	7.2±1.0	16.5±1.3
MUFA	g/day	43.5±6.1	5.0±2.6
PUFA	g/day	16.9±7.5	2.6±3.0
Omega-3 PUFA	g/day	2.4±1.5	0.7±0.1
Omega-6 PUFA	g/day	9.3±6.6	0.6±0.7

%, contribution to the total daily energy intake as per cent; g, grams; kcal, kilocalorie; MUFA, monounsaturated fatty acids; PUFA, polyunsaturated fatty acidsSFA, saturated fatty acids

To capture their habitual dietary intake prior to the study, the participants are asked to complete the European Prospective Investigation into Cancer and Nutrition study Food Frequency Questionnaire (EPIC FFQ; https://www.epic-norfolk.org.uk/about-epic-norfolk/nutritional-methods/ffq/) before their baseline visit. To promote adherence, all study foods are delivered to participants’ homes using a supermarket delivery service, with extra food provided for the evening meals for one other person at home. Participants are provided with booklets (online supplemental appendix 6), with guidance as to how to store and prepare the meals and which additional foods and snacks can be consumed if hungry. The snacks are chosen to ensure they do not affect the MEDAS score of the study arm. To track dietary compliance, participants are asked to record all foods and beverages on the daily checklists in the booklets and provide notes and feedback. Participants are contacted daily to encourage dietary adherence.^31^

**Figure 2:**
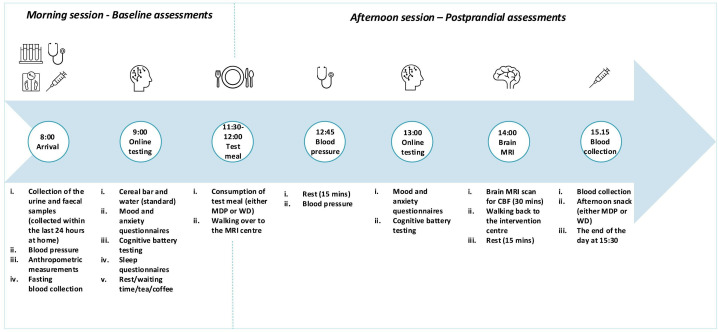
The protocol for the intervention centre visits. On the day 1 visit, participants undergo the full protocol. On day 6 visit, participants undergo the morning session only. CBF, cerebral blood flow; MDP, Mediterranean-style dietary pattern; WD, Western diet.

Participants are asked to visit the intervention centre on day 1 (figure 2), from 08:00 until approximately 15:30. Before their arrival, they are required to collect a urine and faecal sample at home using sample collection kit provided at least 2 days prior to their day 1 visits. The kit includes a stool sample catcher, two plastic tubes with scoop, a biohazard bag, a sealable bag, a urine sample collection pot with a sealable bag, a pair of disposable gloves, an insulated cool bag, two freezer blocks with two sterile outer bags and instructions. They are asked to collect the faecal sample within 24 hours prior to their clinical visit, and the urine sample as the first pass on the morning of their visit (day 1). Participants arrive at the intervention centre in a fasted state (fasted from 20:00 the night before). On arrival, anthropometric (weight and height) and blood pressure (BP) measurements are taken. A nurse collects the baseline blood sample. Participants are then provided with a honey and oat cereal bar. After 15 min rest, participants undergo the mood, anxiety, cognition and sleep testing via the study website; 90 min after completing these tests, participants are served either an MDP or a WD test meal (at 11:30) depending on the arm they are randomised to. Following the meal, participants’ BP is measured at 12:45 and start postprandial mood and cognitive testing at 13:00. At 14:00, they undergo the brain MRI scan and provide a postprandial blood sample at 15:15. Afterwards, participants are provided with an afternoon snack before leaving the unit and consume their day 1 dinner at home. On day 2 morning, participants complete online mood and anxiety testing at home after having a honey and oat cereal bar. On days 2–5, participants complete a sleep diary. An actigraphy is worn throughout the intervention period. On completion of the 5 day intervention, participants return to the intervention centre on the morning of day 6 (08:00–10:00) to repeat the morning assessments, as carried out on day 1 (figure 2).

As menstruation-related hormonal fluctuations can cause disturbance in mood,^32^ neurocognitive functions^33^ and sleep,^34^ a wash-out period of 23-days was chosen to ensure female participants are on the same phase of their menstrual cycle on each intervention arm (ie, 28 days between arm 1 day 1 and arm 2 day 1).

### Outcomes

All outcome measures are summarised in table 4.

**Table 4:** Summary of the outcome measures

	Measurement	Tool used	Time point	Time per measurement point	Location
Screening	Mood	PHQ-9	Prebaseline	9 min	Home
	Anxiety	GAD-7	Prebaseline	6 min	Home
	Initial dietary habits	MEDAS	Prebaseline	10 min	Home
During interventions	Initial dietary profile	EPIC FFQ	Baseline	30 min	Home
	Mood and anxiety	Bond-Lader VAS,POMS	Baseline, postprandial, 24 hours,day 6	30 min	Home (24 hours) and intervention centre (baseline, postprandial and day 6)
	Cognitive functions	NeurOn battery	Baseline, postprandial,day 6	30 min	Intervention centre
	CBF	MRI	Postprandial	30 min	UWWBIC
	Blood pressure		Baseline, postprandial,day 6	5 min	Intervention centre
	Blood samples		Baseline (≥10 hour fasting), postprandial,day 6 (≥10 hour fasting)	15 min	Intervention centre
	Urine and faecal samples		Baseline,day 6		Home collection kits are provided
	Weight and height	SECA scale	Baseline,day 6	5 min	Intervention centre
	Initial sleep profile	PSQI	Baseline	10 min	Intervention centre
	Sleep quality	ActigraphyKSDKSS	Over 5 days	Continuously (for actigraphy)5 min (for KSD and KSS)	Home
	Subjective dietary review score	Non-validated single question	Day 6		Intervention centre
Follow-up	Dietary behaviour	MEDAS	3 months	10 min	Home

CBFcerebral blood flowEPIC FFQEuropean Prospective Investigation into Cancer and Nutrition study Food Frequency QuestionnaireGAD-7Generalised Anxiety Disorder-7KSDKarolinska Sleep DiaryKSSKarolinska Sleepiness ScaleMEDASMediterranean Diet Adherence Screener tool (includes 14-item)PHQ-9Patient Health Questionaire-9POMSProfile of Mood StatesPSQIPittsburgh Sleep Quality IndexUWWBICUniversity of East Anglia Wellcome-Wolfson Brain Imaging CentreVASVisual Analogue Scale

#### Primary outcomes

##### Mood and anxiety

Mood and anxiety levels are monitored using two scales. The primary outcome measure is the Profile of Mood State score (POMS)^35^ with mood also scored using the Bond-Lader questionnaire.^36^ The former has 65 items measuring 6 elements of mood (namely anxiety, anger, confusion, depression, fatigue and vigour); while the latter has 16 items (alert, drowsy, calm, excited, strong, feeble, muzzy, clear-headed, well-coordinated, clumsy, lethargic, energetic, contented, discontented, troubled, tranquil, mentally slow, quick witted, tense, relaxed, attentive, dreamy, incompetent, proficient, happy, sad, antagonistic, amicable, interested, bored, withdrawn, gregarious) under four categories (1. mental sedation or intellectual impairment, 2. physical sedation or bodily impairment, 3. tranquillisation or calming effects and 4. other types of feelings or attitudes) or three mood factors (alertness, contentment and calmness). Both are commonly used in research including three of the four studies included in our systematic review,^25^ allowing a direct comparison of our findings with the limited published literature. The primary outcomes are the ‘contentment’ domain from Bond-Lader and the anxiety domain from POMS.

### Secondary outcomes

#### Cognitive performance

Changes in cognition are assessed using a cognitive battery administered using the NeurOn online platform (https://neuropsychology.online). The following tests are included the following:

1. Reaction Time Test for motor function.
2. Digit Span Test for executive function.
3. Trail Making Test (Trails A and Trails B) for executive function.
4. Sustained Attention to Response Test for executive function and attention.
5. Word Encoding for episodic memory.
6. Word Recognition for episodic memory.
7. Go No-Go for executive function and impulse control.
8. Fragmented Letters Test for visuospatial function.

Attention is an important secondary outcome as it was shown to improve in the short-term in our systematic review.^25^ Attention is measured by the Sustained Attention to Response Task (SART).^37^ In the SART test, participants have a visual presentation of 225 digits on a computer screen in a random order over a 4.3 min period (1150 ms between the onsets of digits) and are expected to respond with a key press except when they see the digit 3.^37 38^ It is a commonly used measure in research and is postulated to be sensitive to everyday attention tasks in traumatic brain injured patients as well as normal (control) individuals.^37 38^

#### Cerebral blood flow

CBF, also known as brain perfusion, can be affected by macronutrient composition^39 40^ and bioactives such as polyphenols,^41^ which are abundant in an MDP. Furthermore, reduced brain energy glucose metabolism and CBF is evident in major depressive disorders^42^ and cognitive decline,^43 44^ which is affected by food intake. An effect of intervention on CBF is proposed to partly underpin the effect of intervention on mood, anxiety and cognitive outcomes. We hypothesise a greater CBF after the MDP meal compared with the WD meal. MRI is considered the gold standard CBF measurement,^41^ with the following sequences used:

1. Time of flight angiography to determine the labelling plane to be used with pseudo-Continuous Arterial Spin Labelling (p-CASL).
2. P-CASL which provides a means of quantifying regional CBF.^45^
3. Magnetisation Prepared Rapid Gradient Echo (MPRAGE) for routine whole brain imaging using rapid acquisition.^46^
4. Fluid-Attenuated Inversion Recovery (FLAIR) to visualise the white matter hyperintensities (WMH).^47^

MPRAGE and FLAIR sequences help to eliminate potential confounders influencing CBF in the present study such as atrophy and WMH.

Resting state functional MRI (rs-fMRI) is used to explore resting neural activity and connectivity between different brain regions including those that are concerned with self-referential processing and salience networks.^48^ During the scan, participants wear a pulse oximeter and respiratory belt to record the influence of cardiac and respiratory processes on measured signal. The scan parameters are taken from the UK-Biobank protocol,^49^ allowing a comparison to this large cohort. Analysis utilises physiological noise modelling, white matter/CSF signal regression and spatial independent components analysis to define resting state networks. Seed-based analysis utilises regions of interest (ROIs) for example, insular cortex, to determine whole brain connectivity. As part of a more extensive analysis, we will employ a Functional Connectivity Multivariate Pattern Analysis approach. This methodology allows us to rigorously test hypotheses across the entire functional connectome as it encompasses all voxel-to-voxel functional connections throughout the entire brain. This exploratory approach complements the seed-based method as it does not require a predetermined parcellation of the brain into ROIs.^50^

#### Blood pressure

Brachial BP is measured with the participant seated and following a 5 min rest period. Measurements are taken using an automatic BP monitor (Omron, 705IT) with an appropriately sized cuff. BP is measured three times and averaged in accordance with published guidelines.^51^

#### Biological samples

Blood, urine and faecal samples are collected at baseline (on day 1) and on completion of the 5 day intervention (on day 6 morning). Postprandial blood samples are collected after the day 1 test meal; 30 mL of blood is collected in three separate tubes (EDTA, Heparin, SST). Several blood biomarkers of mental and cognitive health as well as cardiometabolic health will be assessed including but not limited to plasma glucose, lipids, cortisol and select inflammatory markers and brain-derived neurotrophic factor. On arrival of the day 1 and day 6 mornings, the urine and faecal samples are frozen at −80°C for later analysis.

#### Gut microbial profile

The link between the gut microbiota and anxiety, depression,^52^ and cognition^53^ is evident through the gut–brain axis. Diet composition is an important modulator of microbiome composition and metabolism.^54^ The gut microbiome will be profiled using 16S rRNA Amplicon-based Metagenomic Sequencing of faecal samples.^55^

#### Metabolomics profile

Metabolomics are a tool for providing mechanistic insight into the response to dietary interventions.^56^ The influences of interventions on the metabolomics signature in serum and/or faecal samples will be explored using 1H-NMR-based untargeted metabolomics approach.^57^ Targeted metabolomics by Liquid Chromatography Tandem Mass Spectrometry will be used to measure both straight and branched short chain fatty acids, which are important mediators of gut–brain communication.^58^

#### Sleep timing, quality and quantity and circadian rest-activity rhythmicity

Due to the multidirectional relationship between sleep and circadian disturbances, anxiety and depression,^59^,^61^ cognition including alertness and attention^62^ and food intake,^63^ we will investigate the short-term effects of diet on sleep and circadian rhythmicity. By doing so, we also aim to eliminate the confounding effect of low sleep quality on mood and anxiety. The Pittsburgh Sleep Quality Index (PSQI) will be used to establish the initial sleep profile to detect sleep disturbances on day 0.^64^ Sleep quality is tracked during the two 5 day intervention periods using the Motion Watch 8, which is a wrist-worn actigraphy device. This will allow for the estimation of sleep timing, duration and quality as well as the amplitude and stability of circadian rest-activity rhythmicity known to be interlinked with mental well-being. The Karolinska Sleep Diary (KSD) is a subjective measure and used to estimate the duration, timing and quality of all sleep periods and will complement the actigraphy data to increase the accuracy of the objective sleep quality estimation.^65^ The Karolinska Sleepiness Scale (KSS) is administered every morning during the interventions alongside the KSD, to subjectively measure sleepiness. The KSS is a 9-point scale and asks the user to circle the number that represents the sleepiness level during the immediately preceding 5 min.^66^

#### Dietary behaviour

Participants will be sent the MEDAS questionnaire, 3 months after completing both arms to see if they have made any long-term change to their diets compared with the screening phase. They were also asked to rate how they found following the diets on a scale of 1–10.

### Statistical methods: data collection, management and analysis

#### Sample size calculation

The sample size calculation was based on data from a previous cross-over trial of the effect of MDP adherence in a young healthy adult group.^67^ Assuming an error rate of 0.05 and 90% power, we would require 15 and 20 participants to complete each arm for the primary outcome, which is the contentment, a mood domain from the Bond-Lader scale (9.6 unit expected difference, SD 10.3). To account for up to 20% dropout between random allocation to treatment sequence and study completion, we recruited 25 individuals.

#### Analysis

The main aim of the trial is to test if mood and anxiety can be improved over 5 days of intervention. The primary outcome analysis will use two-way repeated measures analysis with paired analysis taking mean change-scores.

### CBF data analysis steps

1. Data preprocessing: raw data will be converted into the Brain Imaging Data Structure format for standardised data organisation.
2. Structural processing: individual subject-level processing includes structural image processing and segmentation and normalisation to enhance the quality of anatomical data.
3. Single-subject ASL processing: specific processing steps tailored for ASL data will be applied at the individual subject level, including motion correction, registration, partial volume correction and quantification of perfusion.

Group-level analysis: group-level processing through template creation producing a group-average image and subsequent atlas-based ROI statistical analyses.^68^

### Machine learning analysis

Machine learning holds considerable potential for identifying biomarkers and enhancing clinical decision-making in varied contexts and is effective in discerning clinical interventions. Our study will use the Random Forest algorithm to enhance the interpretability of the heterogeneous data. This is a supervised machine learning approach recognised for its adeptness to handle missing values, alleviate data noise and mitigate the risk of overfitting making it a robust choice for our analytical framework.^69^

### Monitoring: incidental findings and adverse events

Measurements that are deemed to be outside the normal clinical range will be reported to GPs as incidental findings. Potential incidental findings may be noted from PHQ-9 and/or GAD-7 questionnaires, blood sample analysis or the MRI scans. Due to the nature of the intervention, that is, commercially available food products, no adverse events are expected. If participants feel in anyway adversely affected by any foods or the principal investigator feels an AE necessitates cessation, the participant will be advised not to continue, and the appropriate measures will be taken. All AE’s will be recorded and handled in accordance with Good Clinical Practice guidelines.

### Patient and public involvement

None.

## Discussion

The MediMood study is an efficacy trial which will provide evidence and mechanistic insights into the acute and short-term effects of an MDP on mental health and cognitive performance in UK adults.

MediMood is the first RCT examining the acute (postprandial up to 5 days) effects of an MDP on mood and anxiety as the primary endpoint. Its strengths are as follows: (1) its controlled intervention design informed by a systematic review,^25^ with standardised meals supported by full food provision and detailed preparation instructions, rather than dietary advice only, (2) its cross-over design,^70^ (3) assessment of several biological mechanisms which are hypothesised to mediate the effects of diet on mental and cognitive health, (4) combination of both objective and subjective assessment/measurement methods, (5) its focus on at-risk individuals for future major psychological and neurological disorders, (6) involvement of adults with no upper age limit as people suffer from mental disorders at every life stage and (7) considers the effects of the menstrual cycle on the study outcomes.

The main limitation of the study is that, to reduce the participant burden, we do not measure mood, anxiety and cognition every day. Second, our MRI scan is in a different location, which causes a delay in the postprandial blood collection. Given the nature of the diets, it is not possible to conduct a double-blinded intervention as participants know which diets they are following which may lead to an ‘expectation bias’.^29^ All clinical data, including MRI and biological samples will be anonymously analysed. Cross-over design requires participants to undergo two interventions which may cause attrition.^70^ However, as our intervention duration is only 5 days, we think it is a low risk for the MediMood study.

Day-to-day low mood, anxiety and poor cognitive performance can adversely affect quality of life for not only those with pre-existing mental and/or cognitive health complaints but also healthy individuals. Therefore, there is a need to identify safe and accessible approaches impacting short-term brain health, which is the focus of the MediMood intervention, which also has a strong mechanistic component. The results will help inform future management strategies and policies for individuals with mental health complaints and in the early stages of age-related cognitive decline.

## Ethics and dissemination

### Research ethics approval

The study has been approved by the London Queen Square, NHS Research Ethics Committee and Health Research Authority (22/LO/0796). Informed consent is provided by all participants in the presence of certified research personnel.

### Dissemination policy

The findings of the study will be disseminated through peer-reviewed publications, conference presentations, public outreach events, local and national news and academic blogs such as www.conversations.com for public members.

### Data deposition

Anonymised data may be made available on request for additional analysis, by contacting AMM (senior author).

## supplementary material

10.1136/bmjopen-2023-082935 online supplemental file 1

## References

[1] World Health Organisation (2022). Mental disorders. Fact sheets. https://www.who.int/news-room/fact-sheets/detail/mental-disorders.

[2] James SL, Abate D, Abate KH (2018). Global, regional, and national incidence, prevalence, and years lived with disability for 354 diseases and injuries for 195 countries and territories, 1990-2017: a systematic analysis for the Global Burden of Disease Study 2017. Lancet.

[3] World Health Organisation (2012). Global burden of mental disorders and the need for a comprehensive, coordinated response from health and social sectors at the country level: report by the secretariat.

[4] NHS Digital (2016). Adult Psychiatric Morbidity Survey: Survey of Mental Health and Wellbeing, England, 2014.

[5] Arias D, Saxena S, Verguet S (2022). Quantifying the global burden of mental disorders and their economic value. E Clin Med.

[7] Cuijpers P, Sijbrandij M, Koole SL (2013). The efficacy of psychotherapy and pharmacotherapy in treating depressive and anxiety disorders: a meta-analysis of direct comparisons. World Psychiatry.

[8] Castaldelli-Maia JM, Scomparini LB, Andrade AG de (2011). Perceptions of and attitudes toward antidepressants: stigma attached to their use--a review. J Nerv Ment Dis.

[9] Simpson CA, Diaz-Arteche C, Eliby D (2021). The gut microbiota in anxiety and depression - A systematic review. Clin Psychol Rev.

[10] World Health Organisation (2021). Comprehensive Mental Health Action Plan 2013-2030.

[11] World Health Organisation (2022). World mental health report: Transforming mental health for all.

[12] Shannon OM, Ashor AW, Scialo F (2021). Mediterranean diet and the hallmarks of ageing. Eur J Clin Nutr.

[13] Bach-Faig A, Berry EM, Lairon D (2011). Mediterranean diet pyramid today. Science and cultural updates. Pub Health Nutr.

[14] Sánchez-Villegas A, Delgado-Rodríguez M, Alonso A (2009). Association of the Mediterranean dietary pattern with the incidence of depression: the Seguimiento Universidad de Navarra/University of Navarra follow-up (SUN) cohort. Arch Gen Psychiatry.

[15] Shafiei F, Salari-Moghaddam A, Larijani B (2019). Adherence to the Mediterranean diet and risk of depression: a systematic review and updated meta-analysis of observational studies. Nutr Rev.

[16] Sánchez-Villegas A, Martínez-González MA, Estruch R (2013). Mediterranean dietary pattern and depression: the PREDIMED randomized trial. BMC Med.

[17] Parletta N, Zarnowiecki D, Cho J (2019). A Mediterranean-style dietary intervention supplemented with fish oil improves diet quality and mental health in people with depression: A randomized controlled trial (HELFIMED). Nutr Neurosci.

[18] Jacka FN, O’Neil A, Opie R (2017). A randomised controlled trial of dietary improvement for adults with major depression (the “SMILES” trial). *BMC Med*.

[19] Bayes J, Schloss J, Sibbritt D (2022). The effect of a Mediterranean diet on the symptoms of depression in young males (the “AMMEND: A Mediterranean Diet in MEN with Depression” study): a randomized controlled trial. Am J Clin Nutr.

[20] Martínez-Lapiscina EH, Clavero P, Toledo E (2013). Mediterranean diet improves cognition: the PREDIMED-NAVARRA randomised trial. *J Neurol Neurosurg Psychiatry*.

[21] Shannon OM, Ranson JM, Gregory S (2023). Mediterranean diet adherence is associated with lower dementia risk, independent of genetic predisposition: findings from the UK Biobank prospective cohort study. BMC Med.

[22] Wu L, Sun DL (2017). Adherence to Mediterranean diet and risk of developing cognitive disorders: An updated systematic review and meta-analysis of prospective cohort studies. Sci Rep.

[23] Jacka FN, Cherbuin N, Anstey KJ (2015). Western diet is associated with a smaller hippocampus: a longitudinal investigation. BMC Med.

[24] López-Taboada I, González-Pardo H, Conejo NM (2020). Western Diet: Implications for Brain Function and Behavior. Front Psychol.

[25] Esgunoglu L, Jennings A, Connole ES (2022). Short-term effects of a Mediterranean-style dietary pattern on cognition and mental well-being: a systematic review of clinical trials. Br J Nutr.

[26] Chan A-W, Tetzlaff JM, Gøtzsche PC (2013). SPIRIT 2013 explanation and elaboration: guidance for protocols of clinical trials. BMJ.

[27] El-Den S, Chen TF, Gan Y-L (2018). The psychometric properties of depression screening tools in primary healthcare settings: A systematic review. J Affect Disord.

[28] Spitzer RL, Kroenke K, Williams JBW (2006). A brief measure for assessing generalized anxiety disorder: the GAD-7. Arch Intern Med.

[29] Shannon OM, Lee V, Bundy R (2021). Feasibility and acceptability of a multi-domain intervention to increase Mediterranean diet adherence and physical activity in older UK adults at risk of dementia: protocol for the MedEx-UK randomised controlled trial. BMJ Open.

[30] Rodríguez-Rejón AI, Castro-Quezada I, Ruano-Rodríguez C (2014). Effect of a Mediterranean Diet Intervention on Dietary Glycemic Load and Dietary Glycemic Index: The PREDIMED Study. J Nutr Metab.

[31] Hall AK, Cole-Lewis H, Bernhardt JM (2015). Mobile Text Messaging for Health: A Systematic Review of Reviews. Annu Rev Public Health.

[32] Akdeniz F, Karadağ F (2006). Does menstrual cycle affect mood disorders?. Turk Psikiyatri Derg.

[33] Symonds CS, Gallagher P, Thompson JM (2004). Effects of the menstrual cycle on mood, neurocognitive and neuroendocrine function in healthy premenopausal women. Psychol Med.

[34] Parry BL, Martínez LF, Maurer EL (2006). Sleep, rhythms and women’s mood. Part I. Menstrual cycle, pregnancy and postpartum. Sleep Med Rev.

[35] McNair DM, Lorr M, Droppleman LF (1971). Manual profile of mood states.

[36] Bond A, Lader M (1974). The use of analogue scales in rating subjective feelings. Br J Med Psychol.

[37] Robertson IH, Manly T, Andrade J (1997). “Oops!”: performance correlates of everyday attentional failures in traumatic brain injured and normal subjects. Neuropsychologia.

[38] Whyte J, Grieb-Neff P, Gantz C (2006). Measuring sustained attention after traumatic brain injury: differences in key findings from the sustained attention to response task (SART). Neuropsychologia.

[39] Page KA, Chan O, Arora J (2013). Effects of fructose vs glucose on regional cerebral blood flow in brain regions involved with appetite and reward pathways. JAMA.

[40] Frank S, Linder K, Kullmann S (2012). Fat intake modulates cerebral blood flow in homeostatic and gustatory brain areas in humans. Am J Clin Nutr.

[41] Roberts SB, Franceschini MA, Silver RE (2020). Effects of food supplementation on cognitive function, cerebral blood flow, and nutritional status in young children at risk of undernutrition: randomized controlled trial. BMJ.

[42] Videbech P (2000). PET measurements of brain glucose metabolism and blood flow in major depressive disorder: a critical review. Acta Psychiatr Scand.

[43] Wolters FJ, Zonneveld HI, Hofman A (2017). Cerebral Perfusion and the Risk of Dementia. Circulation.

[44] Cunnane S, Nugent S, Roy M (2011). Brain fuel metabolism, aging, and Alzheimer’s disease. Nutrition.

[45] Mezue M, Segerdahl AR, Okell TW (2014). Optimization and reliability of multiple postlabeling delay pseudo-continuous arterial spin labeling during rest and stimulus-induced functional task activation. J Cereb Blood Flow Metab.

[46] Brant-Zawadzki M, Gillan GD, Nitz WR (1992). MP RAGE: a three-dimensional, T1-weighted, gradient-echo sequence--initial experience in the brain. Radiology.

[47] Hajnal JV, Bryant DJ, Kasuboski L (1992). Use of fluid attenuated inversion recovery (FLAIR) pulse sequences in MRI of the brain. J Comput Assist Tomogr.

[48] Raimondo L, Oliveira ĹAF, Heij J (2021). Advances in resting state fMRI acquisitions for functional connectomics. Neuroimage.

[49] Stephen M, Smith F-A (2020). UK biobank brain imaging documentation version 1.8.

[50] Nieto-Castanon A (2022). Brain-wide connectome inferences using functional connectivity MultiVariate Pattern Analyses (fc-MVPA). PLoS Comput Biol.

[51] Stergiou G, Palatini P, Asmar R (2018). Blood Pressure Measurement and Hypertension Diagnosis in the 2017 US Guidelines: First Things First. Hypertension.

[52] Foster JA, McVey Neufeld K-A (2013). Gut-brain axis: how the microbiome influences anxiety and depression. Trends Neurosci.

[53] Proctor C, Thiennimitr P, Chattipakorn N (2017). Diet, gut microbiota and cognition. Metab Brain Dis.

[54] David LA, Maurice CF, Carmody RN (2014). Diet rapidly and reproducibly alters the human gut microbiome. Nat New Biol.

[55] Rinott E, Meir AY, Tsaban G (2022). The effects of the Green-Mediterranean diet on cardiometabolic health are linked to gut microbiome modifications: a randomized controlled trial. Genome Med.

[56] Chen L, Zhang J, Teh JPY (2020). Comparative Blood and Urine Metabolomics Analysis of Healthy Elderly and Young Male Singaporeans. J Proteome Res.

[57] Almanza-Aguilera E, Urpi-Sarda M, Llorach R (2017). Microbial metabolites are associated with a high adherence to a Mediterranean dietary pattern using a ^1^H-NMR-based untargeted metabolomics approach. J Nutr Biochem.

[58] Dalile B, Van Oudenhove L, Vervliet B (2019). The role of short-chain fatty acids in microbiota–gut–brain communication. *Nat Rev Gastroenterol Hepatol*.

[59] Alvaro PK, Roberts RM, Harris JK (2013). A Systematic Review Assessing Bidirectionality between Sleep Disturbances, Anxiety, and Depression. Sleep.

[60] Crouse JJ, Carpenter JS, Song YJC (2021). Circadian rhythm sleep-wake disturbances and depression in young people: implications for prevention and early intervention. Lancet Psychiatry.

[61] Salgado-Delgado R, Tapia Osorio A, Saderi N (2011). Disruption of circadian rhythms: a crucial factor in the etiology of depression. Depress Res Treat.

[62] Killgore WDS, Kerkhof GA, Dongen Hpa (2010). Progress in brain research.

[63] Peuhkuri K, Sihvola N, Korpela R (2012). Diet promotes sleep duration and quality. Nutr Res.

[64] Buysse DJ, Reynolds CF, Monk TH (1989). The Pittsburgh sleep quality index: A new instrument for psychiatric practice and research. Psychiatry Res.

[65] Akerstedt T, Hume K, Minors D (1994). The subjective meaning of good sleep, an intraindividual approach using the Karolinska Sleep Diary. Percept Mot Skills.

[66] Miley AÅ, Kecklund G, Åkerstedt T (2016). Comparing two versions of the Karolinska Sleepiness Scale (KSS). Sleep Biol Rhythms.

[67] Lee J, Pase M, Pipingas A (2015). Switching to a 10-day Mediterranean-style diet improves mood and cardiovascular function in a controlled crossover study. Nutrition.

[68] Mutsaerts HJMM, Petr J, Groot P (2020). ExploreASL: An image processing pipeline for multi-center ASL perfusion MRI studies. Neuroimage.

[69] Barberis E, Khoso S, Sica A (2022). Precision Medicine Approaches with Metabolomics and Artificial Intelligence. Int J Mol Sci.

[70] Krogh HB, Storebø OJ, Faltinsen E (2019). Methodological advantages and disadvantages of parallel and crossover randomised clinical trials on methylphenidate for attention deficit hyperactivity disorder: a systematic review and meta-analyses. BMJ Open.

