# Supplementary material for: Acute effect of a Mediterranean-style dietary pattern (MDP) on mood, anxiety and cognition in UK adults with mild to moderate anxiety and depression: the MediMood randomised controlled trial protocol
Source: BMJ Open. 2024 Dec 20;14(12):e082935. doi: 10.1136/bmjopen-2023-082935 (PMC11667456; doi:10.1136/bmjopen-2023-082935)

# **Appendix 1. Mediterranean Diet Adherence Screener (MEDAS) questionnaire**

# ****

# **Appendix 2. MRI Safety Screening Form**


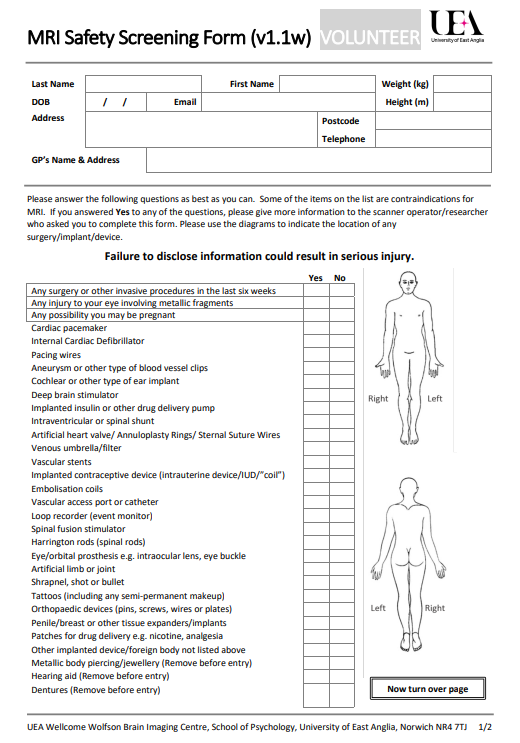


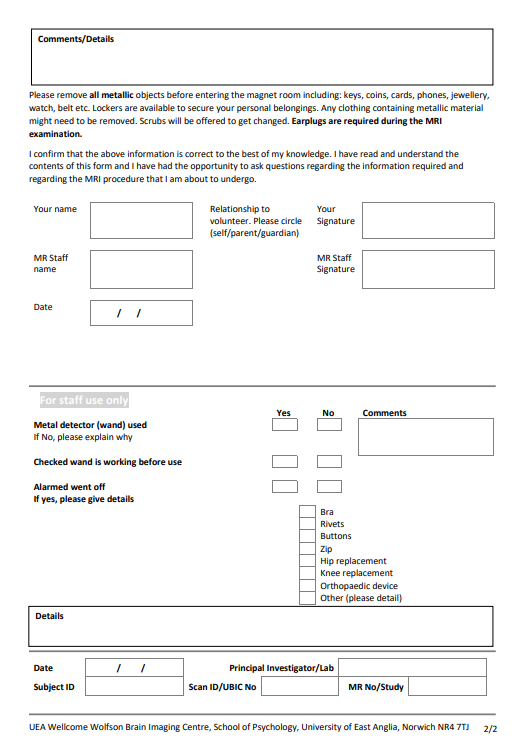


# **Appendix 3. Participant Information Sheet**


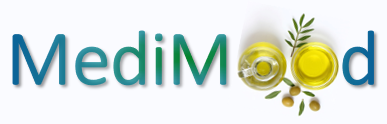


**5-day plant based Mediterranean style and Western style diet on**

**Mood and Anxiety in UK adults**

**PARTICIPANT INFORMATION SHEET**

*13/12/2022, version 2.1, IRAS ID: 320471*

**INVITATION TO PARTICIPATE IN A RESEARCH PROJECT**

We would like to invite you to take part in a research study conducted by the University of East Anglia (UEA). Before you decide to take part, it is important for you to understand why the research is being done and what it will involve. Please take time to read the following information carefully, and do not hesitate to contact us if anything is not clear or if you would like more information.

This study is organised by Professor Anne Marie Minihane (the Principal Investigator) and Miss Latife Esgunoglu.

Members of the study team who can be contacted are:

Miss Latife Esgunoglu and Miss Marrium Liaquat.

To contact the study team:

Tel: +44 (0) 1603591949

Email: [medimood@uea.ac.uk](mailto:medimood@uea.ac.uk)

Before deciding whether or not you would like to take part, you may wish to talk about it with a relative, friend or your local doctor.

If you decide to take part in the research project, you will first be asked to provide consent to participate in the study. You will be asked to sign and return a consent form either electronically or by post which a researcher will check via videoconferencing software (e.g., Zoom). A copy of the signed consent form will be provided to you. By signing it you are telling us that you:

- understand what you have read in this information sheet
- consent to take part in the research project
- consent to have the tests and treatments described
- consent to the use of your personal and health information as described.

***An expression of interest does not commit you to take part.***

**Why are we doing this research and why is it important?**

Mental health issues such as anxiety and depression are common problems worldwide at all stages of life. Although some effective medications and psychotherapies are available to treat depression, these can take time to work, may be expensive, and do not suit everyone.

Scientific evidence shows that what you eat plays an important role on your health, including mental and cognitive (brain function) health. Both a Mediterranean-style diet and a Western-style diet are among the most studied dietary patterns associated with mental health. A Mediterranean-style diet is high in olive oil, fish, fruits and vegetables, nuts, legumes while a Western-style diet is high in processed foods, refined sugar, saturated fat, and salt.

To date, most of the studies examining the impact of diet on mental and cognitive health have been carried out over many weeks or months. We aim to compare the effects of a Mediterranean-style diet and a Western-style diet on mental health and overall quality of life in the short term (over the course of five days)

**What is the MediMood study?**

The MediMood study is designed to explore the short term (5 days) effect of eating a Mediterranean-style diet and a Western style diet on mental and cognitive health in adults. Both diets have been designed by an expert nutrition team. Using dietary profiles based on national intakes in the UK population. The study is funded by MRC-NIHR Nu/Brain Consortium Grant, PhD scholarships of The Republic of Turkiye, and The Commonwealth Scholarship Funds.

**Who can take part in the study?**

We aim to recruit 25 volunteers aged 18 years or over, fluent in English, who have a mild to moderate level of anxiety or depression which we will determine through a questionnaire.

**Unfortunately, you will not be able to volunteer if you:**

- have high levels of depression or anxiety
- are on antianxiety and/or antidepressant medication which is likely to change over the next 3 months
- are already following a Mediterranean diet
- are vegan or vegetarian
- have allergies to any of the study components e.g., nuts or fish
- are not fluent in English language
- are not able to have an MRI scan (e.g., have a pacemaker, suffer from claustrophobia)
- are not prepared to make changes to your diet for 10 days (2 x 5 day periods)
- do not agree your GP to be contacted about your participation and with your screening results.

If you are unsure whether you meet the criteria for our study, please get in touch with the study team and we can talk to you about your suitability.

**Do I have to take part in the study? Can I withdraw after consenting?**

Participation in this research is entirely voluntary. If you decide to take part, you will be free to withdraw at any time and without giving a reason. If you choose to take part, or withdraw from the study, this will not affect your future health care. An expression of interest (for example, contacting the study researchers via telephone or email for further details) does not commit you to participation. If you withdraw, we will keep the data you have given us up to that point.

**Where is the study based? Who is involved?**

The study will take place in the Norfolk and Norwich University Hospitals (NNUH) Clinical Research Facility (CRF) in the Quadram Institute, based on the Norwich Research Park. The study is being conducted by researchers and PhD students at the University of East Anglia (UEA), Norwich. The study also involves research nurses who will take your blood samples.

**What will happen if I agree to take part?**

This study will involve two 5-day dietary intervention study periods blood, urine and faecal samples collected on day one and six of each study periods, computerised psychological and brain function-related tests administered on day one, two and six of each study period, and an MRI scan taken on day one of each study period.

The figures below (on the next page) summarise the stages of the study:

***1. Consent to the study (online, 45 minutes)***

If you are interested in taking part, we will arrange an online videoconference using Zoom (or a phone call, depending on your choice) where a member of the study team will go through the details of the study including dietary instructions with you and answer your questions. When you are satisfied with the information provided and you remain interested in taking part, we will ask you to complete and sign a consent form either electronically or using a print-out version which you can post to the study team. If you prefer, you can take an extra 48 hours to decide to consent and proceed with the study.

***2. Online screening (30 minutes)***

After you consent the study, we will ask you to complete four short questionnaires to establish your eligibility for the study. of the first of these questionnaires will be about your food preferences and how often you currently eat certain foods, and the second and the third will assess certain aspects of your mental health, the final questionnaire will assess your suitability for an MRI scan of your brain. We will also ask you if you are currently on any antidepressant or antianxiety medication. The screening can be done either electronically or paper based, depending on your choice.

Once we have your answers and assess the results, we will compare them to the study inclusion/ exclusion criteria (see information on ‘who can take part in this study?’ above), and if we think you are suitable for the trial, we will contact you via email or telephone to let you know. If you are suitable and happy to proceed, we will then arrange your study dates. If you are not eligible to take part, we will explain why this is, and you will be encouraged to contact your GP if you have any concerns.

**Dietary intervention:** We will ask you to follow a meal plan for 5-days on two different occasions with a four-week break in between. We will provide all your food (we will arrange supermarket delivery to your home) for the two five-day periods and full instructions on how to prepare the food. We will ask you to complete daily checklists (*Appendix 1 and 2*), and we will contact you to find out if you are managing to follow the diet. On days one and six of each of the dietary intervention period, we will ask you to attend on-site appointments at the CRF as detailed below.

***Figure 1.*** The study overview


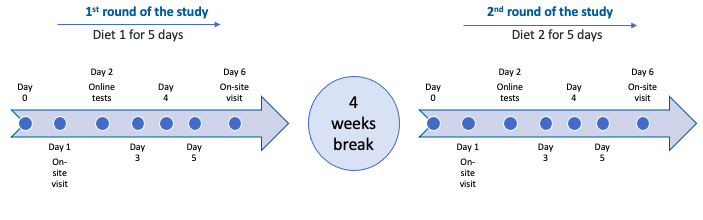
***Figure 2.*** The study steps

***
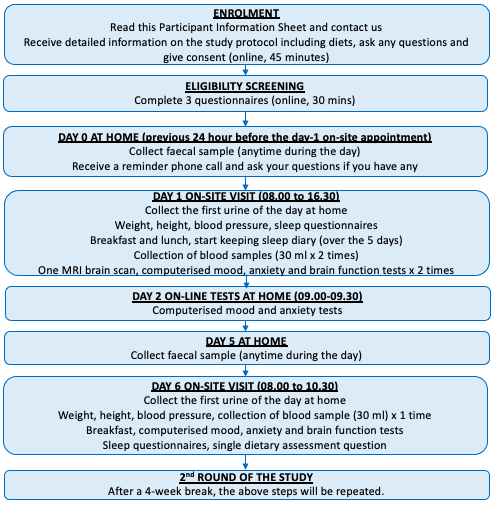
***

***3. On-site and online measurement appointments***

**Day 0:** In the 24 hours prior to the day-1 visit, you will be asked to collect a faecal sample using the kit and instructions sent to you beforehand. You will be asked not to eat or drink (except water) from 8 pm until you arrive for your on-site appointment the following morning at 8 am.

**Day 1 (8 hours):** You will be asked to collect the first urine you pass in the morning at home in a container provided. You will be invited to the CRF, UEA, at 8 am in the morning. During your visit, a researcher will take your urine and faecal samples collected at home, measure your weight, height, and blood pressure, and collect a blood sample (30 ml).

You will be then provided with a light breakfast of a cereal bar and water. Following a 10-15 minute break, we will ask you to complete mood-related and cognitive tests with a researcher. These tests will assess different aspects of your mood and overall brain function and will take approximately one hour. We will then ask you to remain in the CRF and rest for 2 hours and complete a short questionnaire assessing your usual sleep habits.

At approximately 12 pm, we will provide you with a lunch and ask you to visit the main UEA campus where you will have a brain scan using MRI which will take approximately 30 minutes and allow us to look at the structure, function, and blood flow of your brain. As the MRI scanner has a strong magnetic field, it is not safe for some people to have an MRI scan. If you have any concerns about the scan e.g., metal pins (used to repair fractures) or other implemented medical devices, please bring this to the attention of the researcher (please see Appendix 3 for MRI specific information later in this document). Subsequently, we will repeat the mood and cognitive tests and take you back to the CRF and where a nurse will take another blood sample (30 ml), at around 4.30 pm.

**Day 2 (30 mins):** We will ask you to complete online mood and anxiety tests at home. Similar to day one, first we will ask you to have the light breakfast supplied (a cereal bar and water), and then complete (9-9.30 am) the online tests via a link which will be sent to you. After completing the tests, you will follow your assigned meal plan including breakfast.

**Day 5:** Similar to day 0, we will ask you to collect a faecal sample at home. You will be asked not to eat or drink (except water) from 8 pm until you arrive for your clinical visit the next day.

**Day 6 (2.5 hours):** You will be asked to collect the first urine sample of the day in a container provided. We will invite you to the CRF for the last time before your 4 weeks rest period. As for Day 1,we will ask you to arrive at the CRF fasted at 8 am. We will measure your weight and blood pressure and a nurse will collect a blood sample (30 ml), along with the already collected urine and faecal samples. We will provide the same light breakfast and ask you to complete mood and cognitive tests (1 hour), a sleep diary and ask how you found following the meal plan.

**Sleep assessment:** As sleep plays a significant role on our mood and brain functions and is affected by what we eat. Therefore, we will measure your sleep quality throughout the two 5-day intervention periods.

You will be asked to complete a questionnaire on your day 1 on-site visit for us to establish your sleep quality (5 mins). You will also be required to keep a sleep diary (*Appendix 4*) covering each 5-day intervention period. You will be asked to estimate the duration, timing, and quality of all sleep periods (3 mins).


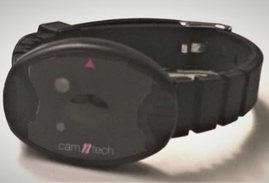


You will be asked to wear a MotionWare 8 actiwatch (CamNtech Ltd, UK) continuously during the two 5-day intervention periods (24 hours per day) (Figure 3). Actiwatches are compact, unobtrusive, lightweight, water-resistant wrist worn devices measuring sleep rhythms. We will not hold you responsible if watches become broken or lost when they are with you. In case of broken/lost devices, please contact us for a replacement.

Figure 3. Image of a MotionWare 8 actiwatch

We will ask you to not to change your physical activity levels and use of probiotic supplements use over the course of the study, as these could affect your study results. After the completion of your first 5 days period, you will have a 4 week break where you will continue to follow your usual diet. We will then invite you for to start the second diet for five days and attend the same on-site appointments as for the first phase. This time you will allocated to the other diet, for example, if you were on a Mediterranean diet in the 1^st^ round, you will be following a Western diet in the 2^nd^ round.

**How many times will I need to come to the clinical research facility?**

You will be expected to visit us 4 times in total over the course of two months.

**What if any abnormal results emerge?**

MediMood is not a clinical examination and we do not run diagnostic tests. It is important to note that elevated scores of mental health screening are not a clinical diagnosis of depression or anxiety.

However, if any of your screening tests (mental health questionnaires), and/or MRI scan are outside of the range considered healthy, we will provide signposting to relevant services where you can access further support (for mental wellbeing support) and contact your GP who may then contact you to discuss your results further. Please note that a research MRI scan is not a substitute for proper clinical investigation, if you suspect you are suffering from a neurological problem (affecting the brain or nervous system) you should always consult your GP in the first instance.

**What will be measured in the samples collected?**

The blood samples collected during your assessment visits will be analysed for blood glucose, fats, markers of brain function, vascular health and inflammation, hormones, levels of antioxidants, and select gene variants (from your DNA).

We will analyse your urine and faecal samples for various markers of gut and brain health. In the faecal samples we will also assess your gut bacteria as recent evidence suggests that gut bacteria are important in overall health, including mental wellbeing.

The samples collected will be securely stored in the biorepository, for up to 10 years, which is only accessible by approved personnel with swipe card access. These samples will be de-identified and only have your study ID on them.

**Are there any risks or burdens associated with taking part in the study?**

Both diets are based on commercially available foods which are already widely consumed by public. There is a small risk that change in your diet may affect your mood/anxiety levels. If you experience a change in your health (including your mood and anxiety) during the study and you are concerned please contact a member of the study team who will immediately signpost you to services that can offer support. If any concerns about the psychological wellbeing of any participant are raised, the Consultant Clinical Psychologist attached to the research team will be contacted for advice. We may also ask you to stop following the diet.

If food and diet are sensitive subjects for you, you may want to consider whether participation in MediMood is right for you.

It is normal that you feel some mild discomfort when giving a blood sample and there is a risk of slight tenderness on your arm and occasional bruising. The nurses involved in our study are specifically trained at taking blood samples.

There is also some risk of becoming tired or frustrated when completing the questionnaires and/or cognitive tests, and of symptoms of cognitive decline being identified due to participating in the research. However, researchers will provide you as many breaks as you need.

With the appropriate safety checks in place MRI is a safe, non-invasive imaging technique, with no known side effects. There are no obvious risks from the scans that you will be asked to undergo. If you become tired or uncomfortable during the MRI scan, please let the MRI operator know and we can take a break or end the session.

**Where can I get mental wellbeing support?**

If you need support with your wellbeing or mental health, there are many services available which are ready to support you.

Call your GP at first instance. If you are a student or a staff member at UEA, you can also contact the university wellbeing services. There are also several charities available for support including Mind and Samaritans who have volunteers to listen to you through helplines/nightlines. Please find the contact details below:

UEA Medical Centre: 01603 251600

UEA Student Support Service: [studentsupport@uea.ac.uk](mailto:studentsupport@uea.ac.uk)

UEA Counselling Service: [csr@uea.ac.uk](mailto:csr@uea.ac.uk)

Norwich Nightline: Call on 01603 597298, or text on 07794 924366

Wellbeing: Call on 0300 123 1503 or email to [admin@wellbeingnandw.co.uk](mailto:admin@wellbeingnandw.co.uk)

Mind (Norwich branch): 0300 330 5488 or email: enquiries@norfolkandwaveneymind.org.uk

Samaritans: Free phone 116123 (24-hour service) or email jo@samaritans.org

**What are the potential benefits of taking part?**

Your efforts with contributing to the study will contribute to a better understanding of how diet affects mental wellbeing in the short term. This will inform policy makers and contribute to future dietary recommendations focussed on improved mental health.

**How will we use information about you?**

We will need to use information from you for this research project.

This information will include your

- Initials
- Name
- Contact details

People will use this information to do the research or to check your records to make sure that the research is being done properly.

People who do not need to know who you are will not be able to see your name or contact details. Your data will have a code number instead.

We will keep all information about you safe and secure.

Some of your de-identified research results, i.e., you cannot be identified, will be shared with national and/or international scientific colleagues for further scientific analyses. These colleagues must follow our rules about keeping your information safe.

Once we have finished the study, we will keep some of the data so we can check the results. We will write our reports in a way that no-one can work out that you took part in the study.

**What are your choices about how your information is used?**

- You can stop being part of the study at any time, without giving a reason, but we will keep information about you that we already have.
- We need to manage your records in specific ways for the research to be reliable. This means that we won’t be able to let you see or change the data we hold about you.
- If you agree to take part in this study, you will have the option to take part in future research using your data saved from this study.
- If you were to lose capacity to verbally consent to your continued participation in the research you would no longer be able to participate, however we would retain and use any collected data.

**Where can you find out more about how your information is used?**

You can find out more about how we use your information

- by asking one of the research team
- by sending an email to the University’s Data Protection Officer, Ellen Paterson at: [dataprotection@uea.ac.uk](mailto:dataprotection@uea.ac.uk) or
- by ringing the University’s Data Protection Officer on 07824 527234.

**What will happen to the results of the research study?**

The results of this research study will be published in scientific journals and presented at national and international scientific meetings. All results will be in an anonymised format so that no individual can be identified. The information will only be used for the purpose of health and care research and cannot be used to contact you or to affect your care. It will not be used to make decisions about future services available to you, such as insurance. Once the data has been made public, we will send you a letter summarising our findings. Unfortunately, we cannot report on the findings of specific individuals.

**Are there any circumstances in which you would divulge my Personally Identifiable Information to anyone outside of the research team?**

We have a duty of care to volunteers and the general public. If you give us information that indicates a real risk of harm to yourself or another person, we have a responsibility to share that information with relevant services. You should only consent to taking part if you understand this possibility.

**Expense Payments**

Participating in this study is on a voluntary basis. Participants will receive a one-off payment of £50 in the form of a gift voucher, to cover costs incurred. This will be provided by a member of the research team upon completion of the 2x5 days study period. You can claim travel expenses to the NNUH CRF. All study food will be provided.

**Who has reviewed the study?**

The study is sponsored by the UEA and has been reviewed and received a favourable opinion from Health Research Authority and the associated [insert the name of approving ethics committee] Research Ethics Committee who works independently and have a duty to protect research volunteers’ safety, rights, wellbeing, and dignity.

**Insurance**

Insurance for the study is from the NNUH for the clinical activities undertaken in the CRF and from the UEA for all other activities.

**What if I want to complain?**

If you have any concerns about the study and your participation in it, or wish to make a complaint, please contact Professor Charles ffrench-Constant, pro-vice-chancellor for the Faculty of Medicine and Health Sciences (cffc@uea.ac.uk).

**Contact for further information**

Thank you for reading this and showing an interest in our study. If you have any questions or would like further information about the study, please contact the study team who are happy to answer your questions.

Phone: +44 (0) 1603591949

Emails: [medimood@uea.ac.uk](mailto:medimood@uea.ac.uk)

# **Appendix 4. Consent Form**


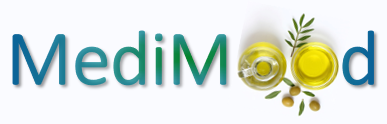


**MediMood: A randomised controlled trial investigating the immediate effects of a plant based Mediterranean-style dietary pattern (MDP) and a Western-style diet on mood, anxiety and cognition in adults with mild to moderate mental health complaints**

*Study IRAS ID: 320471*

Principal Investigator: Prof Anne Marie Minihane

**I consent to participate in the project named above.** I have been provided a copy of the participant information sheet and this consent form (dated 24/05/2023 and version 2.3) and any questions I have asked were answered to my satisfaction.

***Please initial to confirm acceptance***

I agree to be interviewed by a researcher and complete questionnaires asking about

my medical history, sleep, mood and anxiety states, and diet

I agree to make changes to my diet for 10 days as part of the research and receive to

supermarket deliveries of foods

**During the 10 days intervention period, I also agree to:**

*attend four clinical visits to participate in a series of mood, and brain function tests and*

*have my height, weight and blood pressure measured*

*have an MRI brain scan which is a research scan, not a clinical scan and will not*

*be explicitly screened for medical disorders or diagnostic purposes.*

*provide a venous blood sample at the four clinical visits (which will be used for research*

*purposes to assess blood glucose, fats, hormones, markers of brain function, genetics,*

*vascular health, inflammation, levels of antioxidants)*

*provide urine and faecal samples at home prior to the four clinical visits (which will be*

*used for research purposes to assess markers of gut and brain health and gut bacteria)*

*take part in two tests of mood at home*

*complete a daily dietary checklist*

*wear an actiwatch continuously during the two 5-day intervention periods*

*(for 24 hours per day)*

*complete a dietary questionnaire 3 months after the intervention*

I agree for my GP to be contacted if

*My mental health screening indicates above a low level of anxiety or depression*

*A significant abnormality is noticed in my blood results or MRI scan by the*

*University of East Anglia Wellcome-Wolfson Brain Imaging Centre (UWWBIC)*

Please provide your GP details below:

**GP name:**

**GP address & postcode:**

I agree for my data/samples to be used for future studies for up to 10 years which have been

approved by the ethics committee

I understand that one copy of the signed version of this form will be provided to me and

one copy will be kept for the research team’s record.

*I acknowledge that:*

1. *the possible risks have been explained to me to my satisfaction*
2. *my participation is voluntary and that I am free to withdraw from the project at any time*
3. *this project is for the purpose of research and not for profit*
4. *any personal or health information about me which is gathered in the course of my participation*

*will be treated as highly confidential, and retained and analysed solely for the purposes of the study*

1. *my anonymity will be preserved, and I will not be identified in publications or otherwise without*

*my express written consent.*

1. *UEA has a duty of care to volunteers and the general public, therefore, in case of any possibility of harm to myself or others, my personally identifiable information may be divulged.*

I agree to be contacted about future studies

*(Please note that this is optional – only initial if you are happy to be contacted for*

*future studies)*

By signing this document, I agree to the above statements and to participate in this project.

**Name of Participant:**

**Signature Date**

**Name of Investigator:**

**Signature Date**

# **Appendix 5. Meal plans**

**2.1. The 5-day meal plan of the Mediterranean diet arm**

|  | **Day 1** | **Day 2** | **Day 3** | **Day 4** | **Day 5** | **List of allowed snacks** |
| --- | --- | --- | --- | --- | --- | --- |
| **Breakfast** | Honey and oats cereal bar 42g | Honey and oats cereal bar 42g (before testing)  Greek style yoghurt 110g  Blueberries 80g | 2 boiled eggs  1 slice of wholemeal bread 40g | Greek style yoghurt 110g  Blueberries 80g  Walnuts 30g | Greek style yoghurt 110g  Blueberries 80g  Walnuts 30g | - Rice cakes, oat cakes, wholemeal bread, toast  - Cottage cheese or low-fat cream cheese spread  - Low sugar breakfast cereal with milk  - A small glass of wine (125ml/day)  - Sugar free drinks |
| **Morning snack** | NA | Orange juice 150ml | Orange juice 150ml | Orange juice 150ml | Orange juice 150ml |  |
| **Lunch** | Salad with salmon and lentils  *- Salmon 170g*  *- Green lentils 125g*  *- Tomato 140g*  *- Lettuce 100g*  *- EVOO 25ml*  *- Lemon juice 1 tbsp*  *- Balsamic vinegar 1 tbsp*  Walnuts 30g  Orange juice 150ml | Falafel salad  *- Falafel balls 150g*  *- Houmous 30g*  *- Cucumber 60g*  *- Mixed salad leaves 40g*  *- Cherry tomatoes 90g*  *- EVOO 25ml*  *- Balsamic vinegar to taste* | Lentil and turmeric daal 400g/1 pot  EVOO 25ml | Baked potato with tuna and salad  *- Canned tuna, drained 105g*  *- Cucumber 90g*  *- Cherry tomatoes 105g*  *- EVOO 25ml*  *- Balsamic vinegar to taste* | Pasta and tomato sauce  *- Wholemeal pasta 75g*  *- Red onion 75g*  *- Garlic 6g*  *- Canned tomatoes 200g*  *- Red pepper 80g*  *- EVOO 25ml* |  |
| **Afternoon snack** | Banana* | Houmous 30g  Cucumber 90g | Walnuts 10g  Pear* | Figs 30g | Houmous 60g  Cucumber 90g  Carrot sticks 80g |  |
| **Dinner** | Chilli bean soup 300g  EVOO 25ml  2 slices of wholemeal bread 80 g | Black bean and butternut squash chilli  *- Frozen spinach blocks 80g*  *- Frozen butternut squash cubes 125g*  *- Red onion 75g*  *- Tinned tomatoes 200g*  *- Black beans 120g*  *- Chilli pepper 10g*  *- Tomato puree 10g*  *- Garlic 3g*  *- Coriander 15g*  *- EVOO 25ml* | Salmon, new potatoes, salad  *- Salmon 1 fillet*  *- Pesto 24g/1tbsp*  *- New potatoes, boiled 85g*  *- Mixed salad leaves 40g*  *- Cherry tomatoes 90g*  *- Garlic 3g*  *- EVOO 25ml* | Grilled chicken with Mediterranean vegetables and couscous  *- Grilled chicken breast 1 fillet*  *- Butternut squash chunks 125g*  *- Frozen sliced mixed peppers 125g*  *- Wholewheat couscous 50g*  *- EVOO 25ml* | Chicken with chickpeas in tomato sauce  *- Chicken 1 fillet*  *- Red onion 75g*  *- Red pepper 160g*  *- Garlic 6g*  *- Canned tomatoes 200g*  *- Canned chickpeas 120g*  *- Spinach 80g*  *- EVOO 25ml* |  |
| **Evening snack** | Fruit salad 200g | Walnuts 10g  Figs 30g | Mixed nuts 25g  Prunes 205g | Mixed nuts 25g  Pear* | Apple*  Pear* |  |

Note: If the ingredients are not listed underneath, it is a ready-to-eat food.

*: Loose fruits

EVOO: Extra virgin olive oil

**2.2. The 5-day meal plan of the Western diet arm**

|  | **Day 1** | **Day 2** | **Day 3** | **Day 4** | **Day 5** | **List of allowed snacks** |
| --- | --- | --- | --- | --- | --- | --- |
| **Breakfast** | Honey and oats cereal bar 42g | Honey and oats cereal bar 42g (before testing)  1 slice of white bread 40g  Jam 18g  Butter 12g | Frosties cereal 50g  Semi skimmed milk 135g | 2 fried eggs  2 sausages  Butter 12g  1 slice of white bread 40g | Frosties cereal 50g  Semi skimmed milk 135g | - White bread, toast with jam or marmalade (no butter)  - High sugar breakfast cereal with milk  - Chips  - Crisps  - No fruit juice  - No wine (other alcoholic drinks allowed if they wish to do so) |
| **Morning snack** | NA | KitKat 2 fingers 20.7g | KitKat 2 fingers 20.7g | KitKat 2 fingers 20.7g | KitKat 2 fingers 20.7g |  |
| **Lunch** | Ham roll  *- Roll*  *- Oak wafer thin ham 125g*  *- Mayonnaise 12g*  *- Butter 14g*  Cola 330 ml  2 x Mars bars 39.4g | Quiche Lorraine 160g/1 quiche  Potato salad 100g | Bacon roll  *- Bacon, grilled 150g*  *- Bun, 1 roll 63g*  *- Butter 12g*  *- Ketchup 24g* | Cream of chicken soup 400g  2 slices of white bread 80g | Quiche Lorraine 160g/1 quiche  Crisps 22g/1 pack  Cola 150ml |  |
| **Afternoon snack** | Twix bar 20g | Cola 150 ml | Bakewell tart 46g | Chocolate mousse 59g/1 pot | Bakewell tart 46g |  |
| **Dinner** | Cream of chicken soup 400g  1 slice of white bread 40g  Butter 6g | 2 sausages, grilled/oven baked  Chips 165g  Ketchup 24g | Meaty pizza 157g  Potato salad 100g  Cola 150 ml | Spaghetti carbonara 400g  Cola 150 ml | Burger and chips  *- Beef burger 114g*  *- Bun 63g/1 roll*  *- Butter 12g*  *- Chips 135g*  *- Ketchup 24g* |  |
| **Evening snack** | Bakewell tart 46g | KitKat 2 fingers | Vanilla and chocolate cheesecake 90g | Cadbury caramel cake bars 50g | Chocolate mousse 59g |  |

Note: If the ingredients are not listed underneath, it is a ready-to-eat food.

# **Appendix 6. Dietary booklets providing instructions for participants**


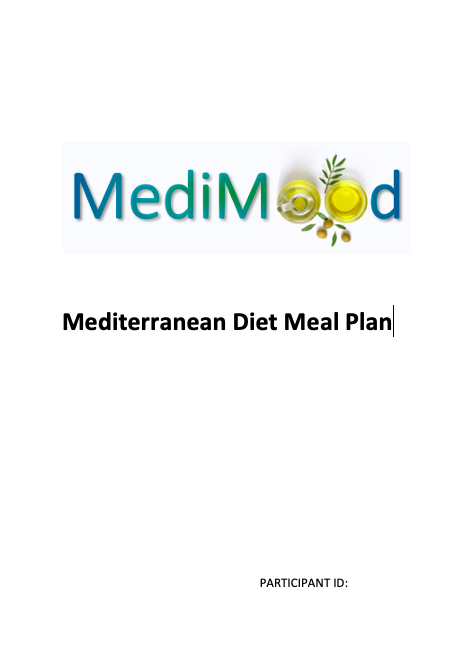


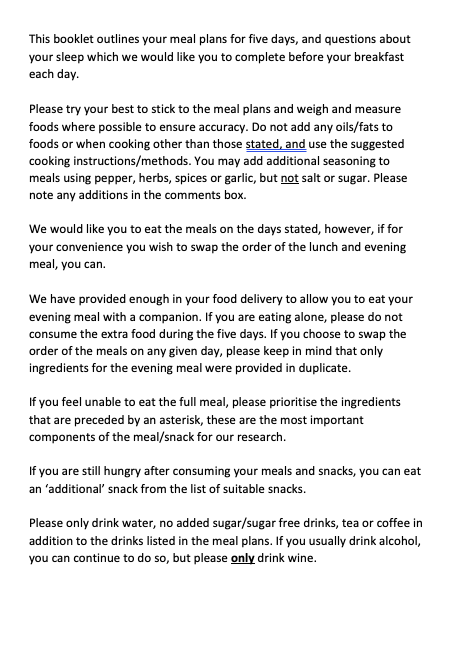

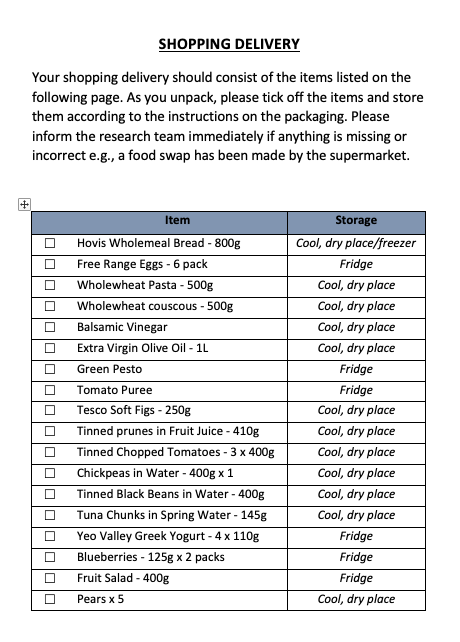

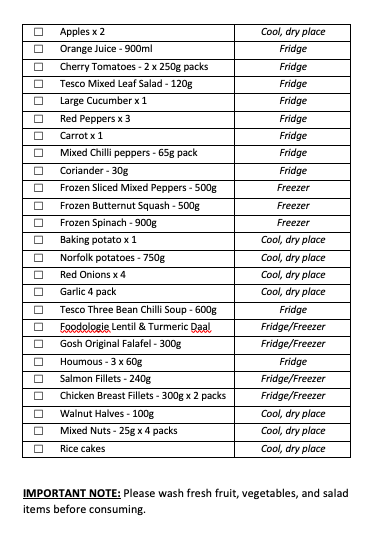

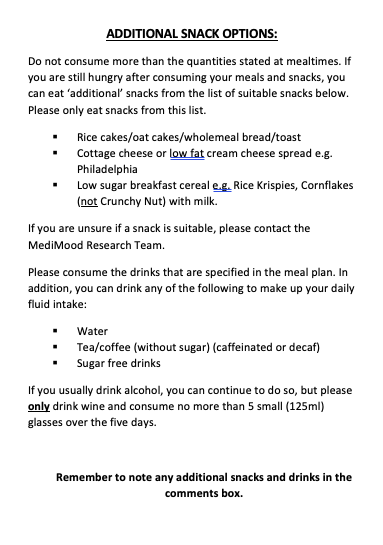

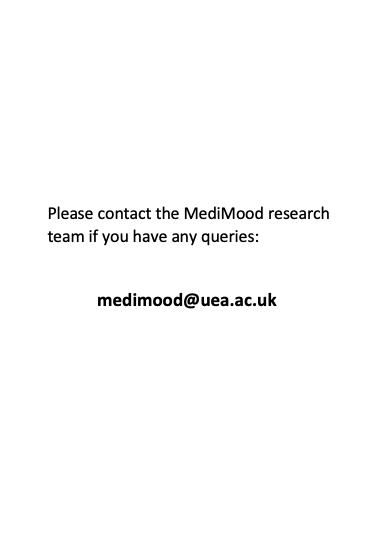


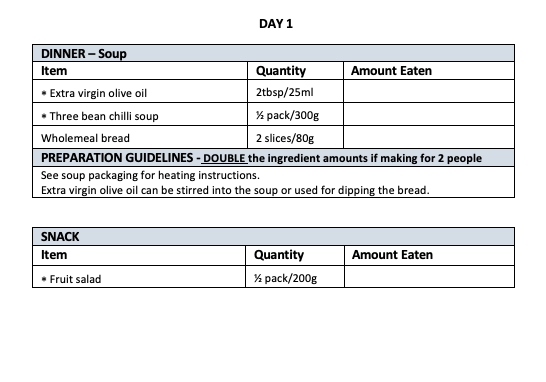


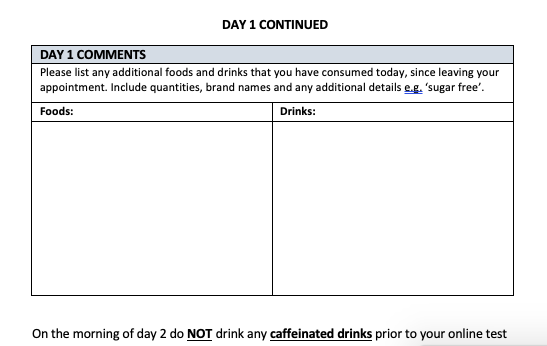

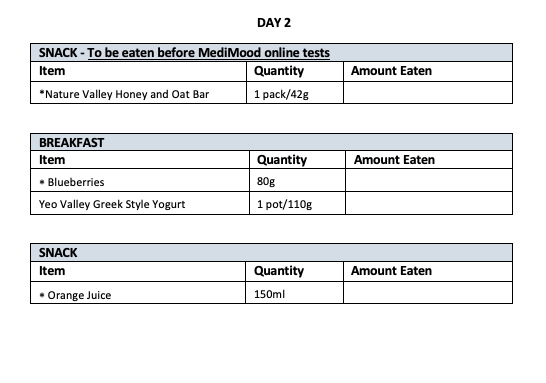

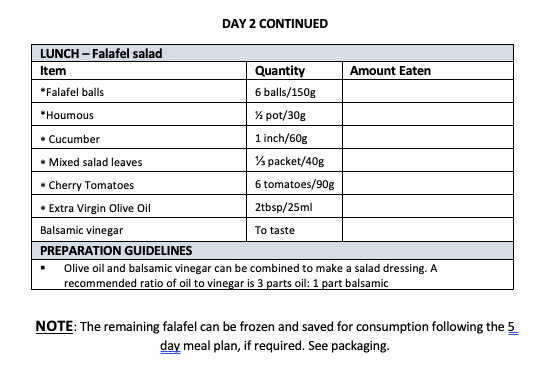

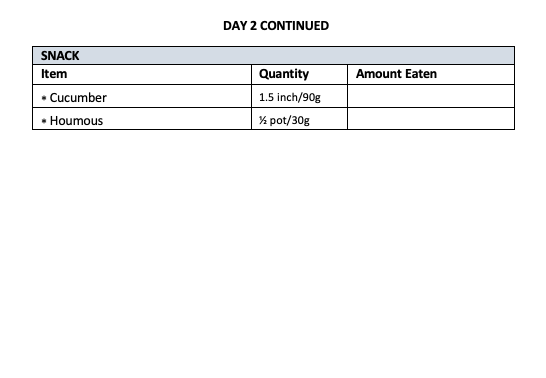

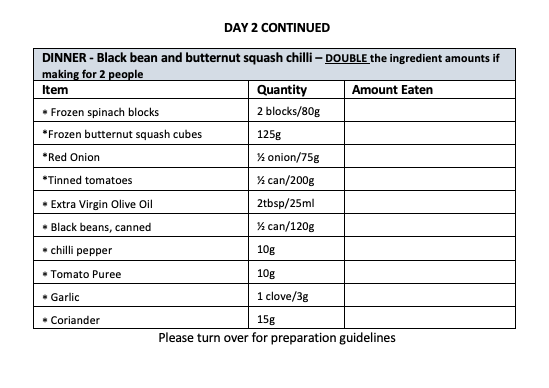

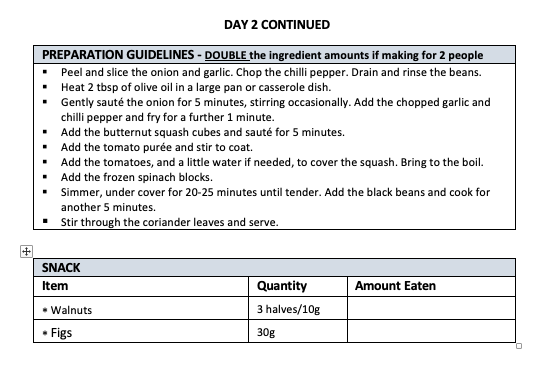

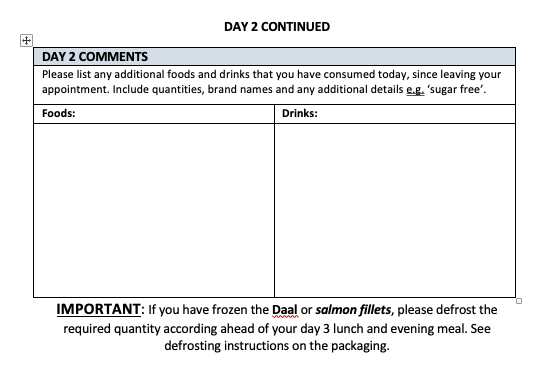

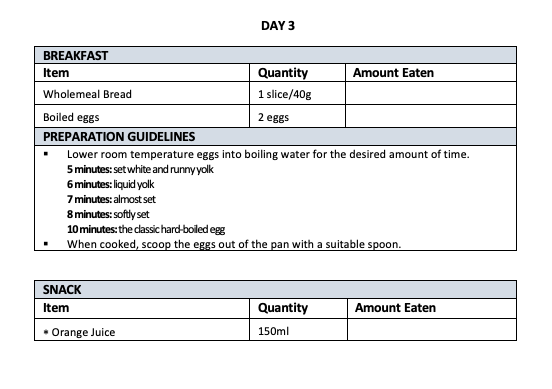

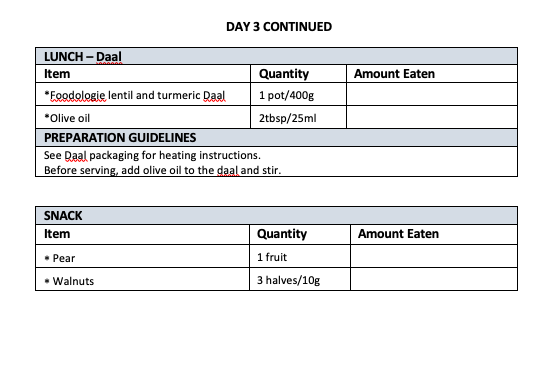

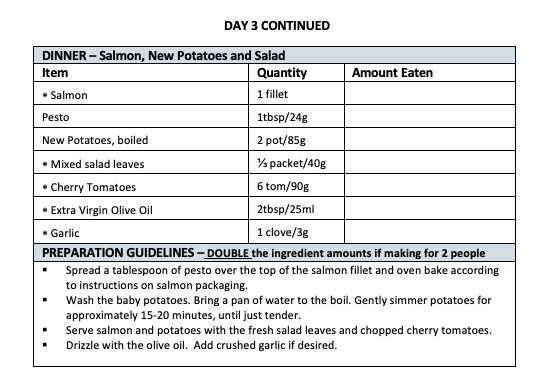

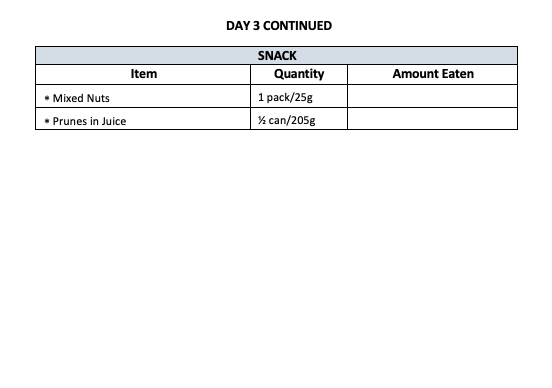

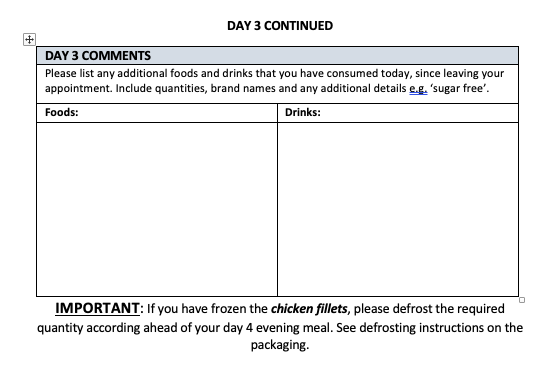

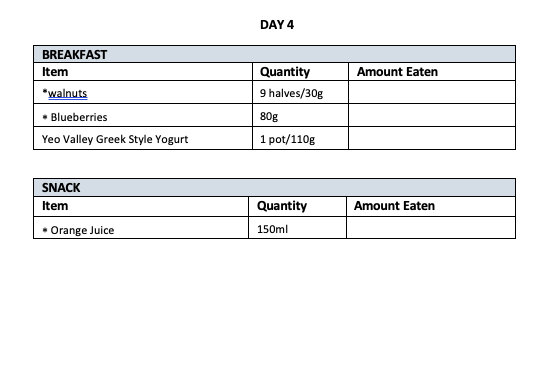

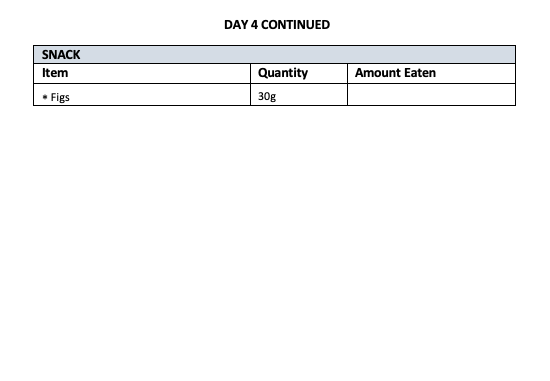

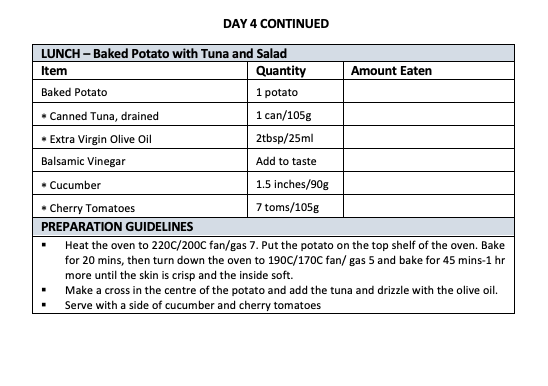

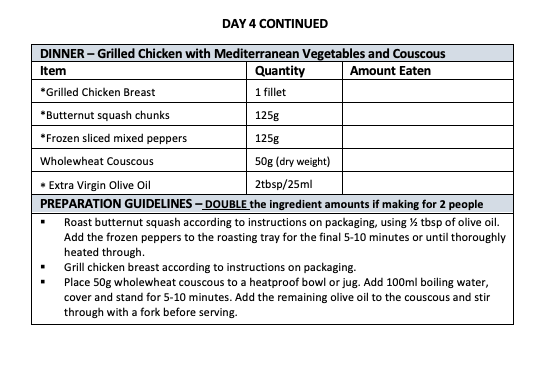

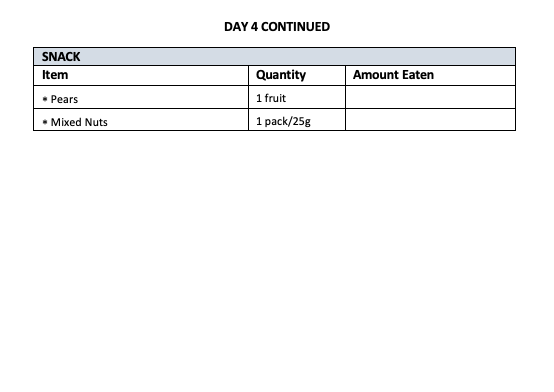

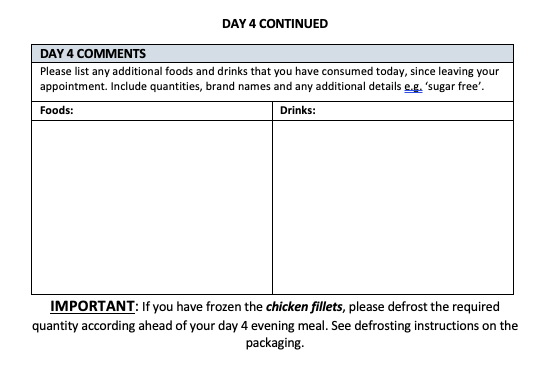

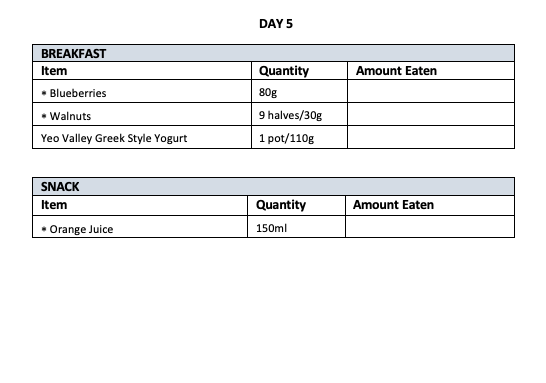

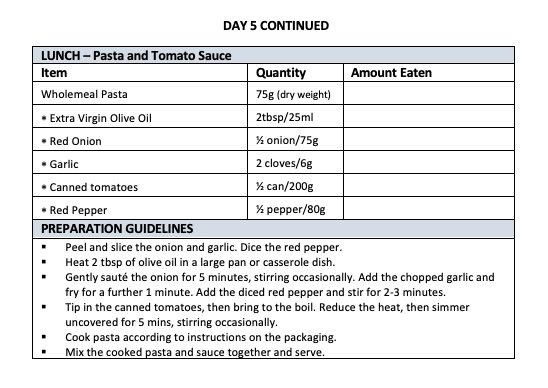

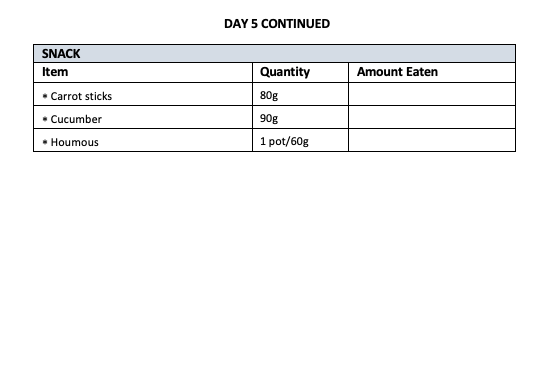

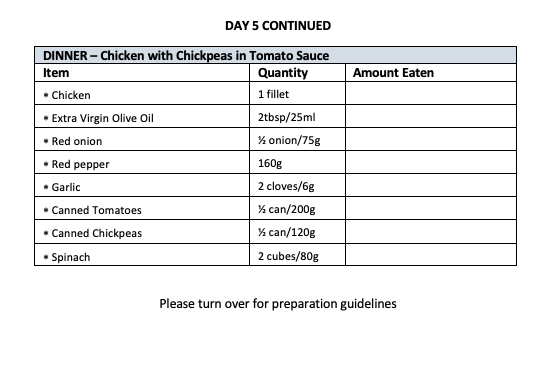

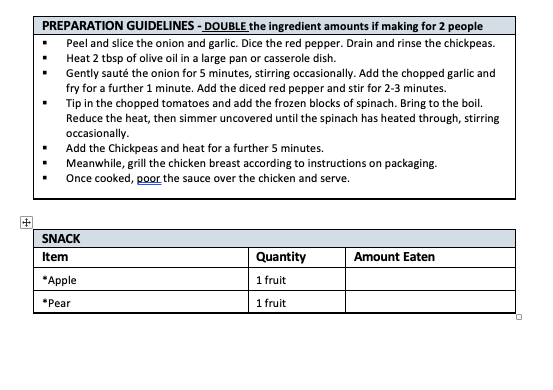

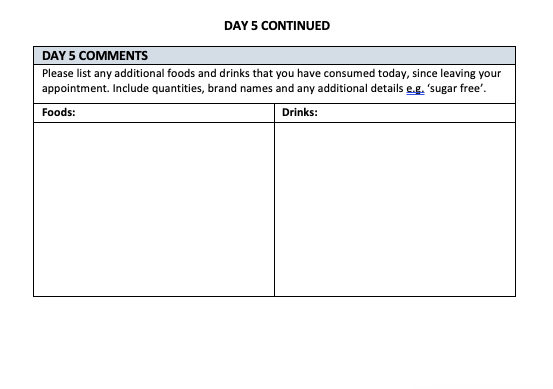


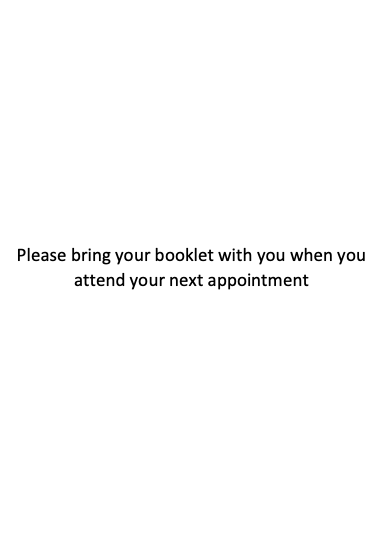


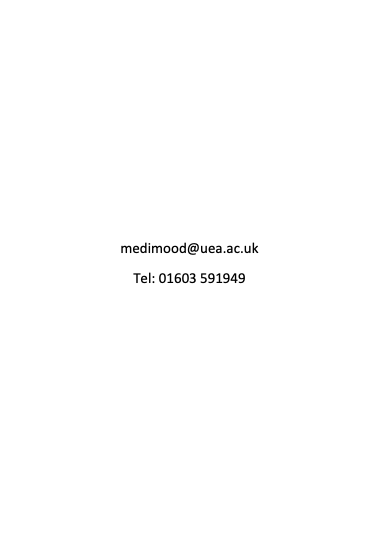


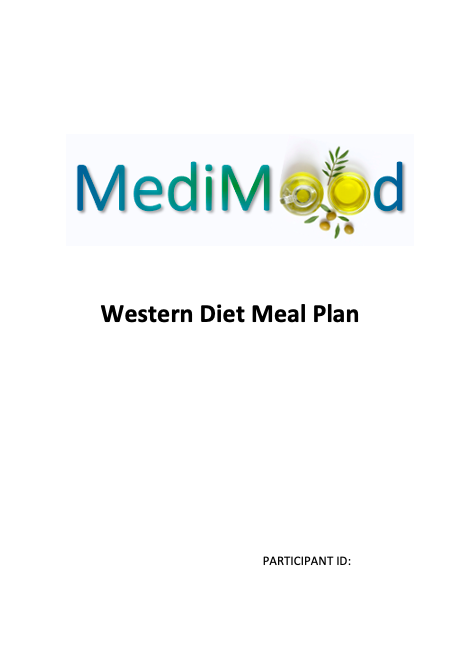

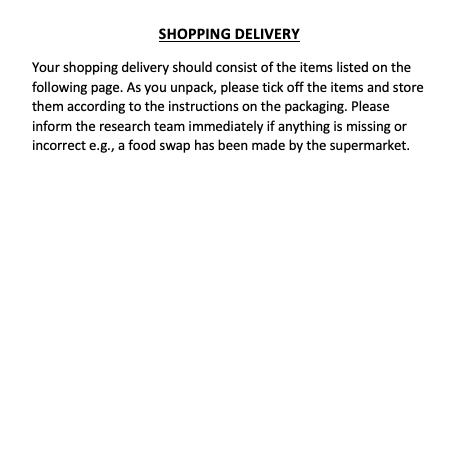

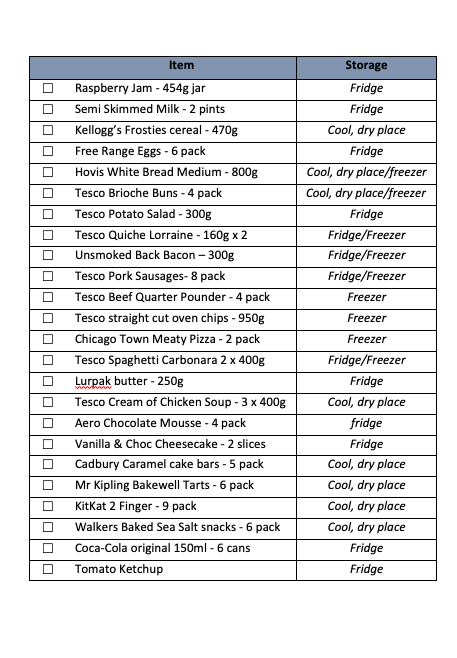

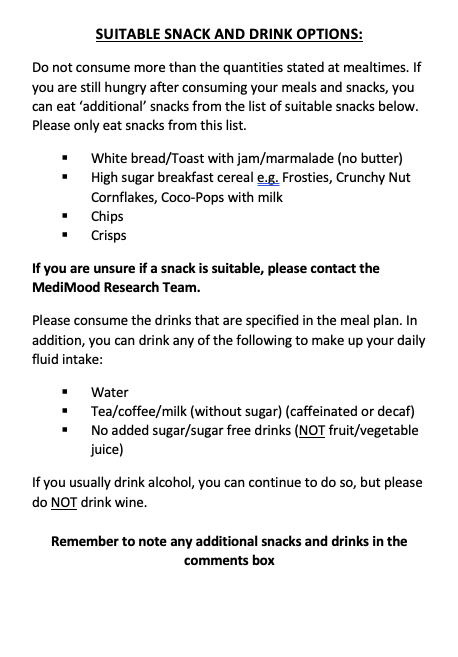

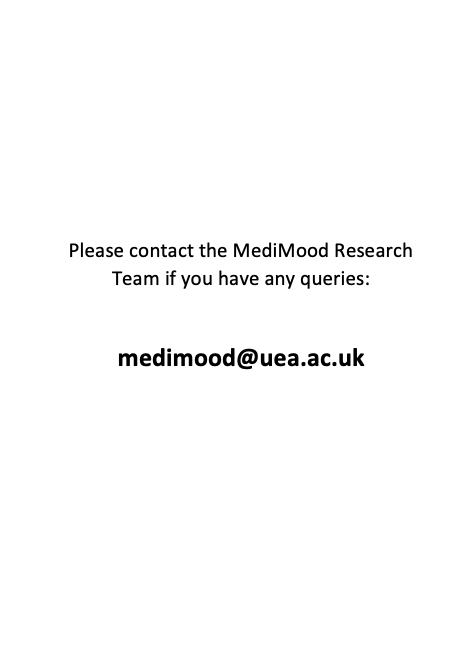


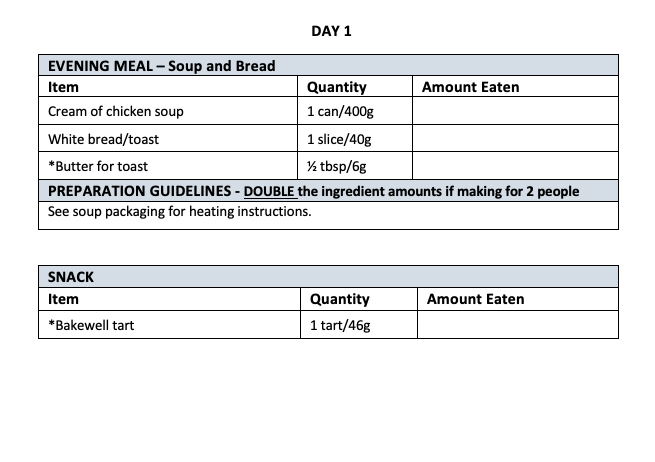

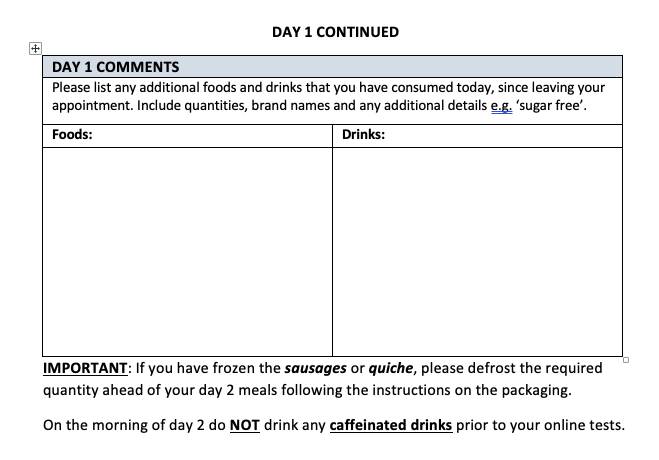

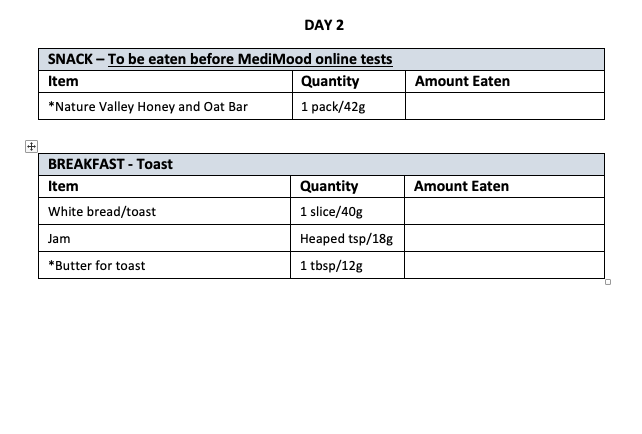

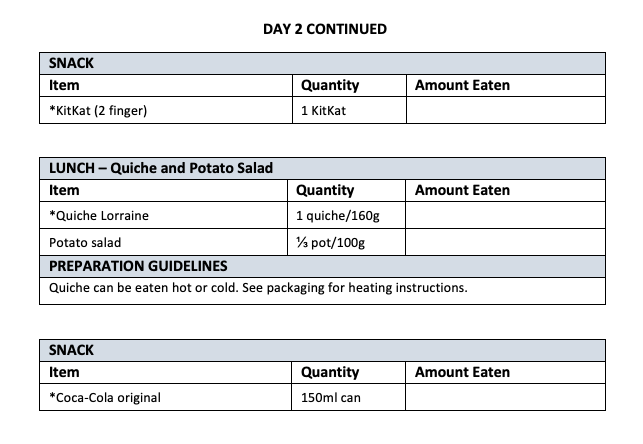

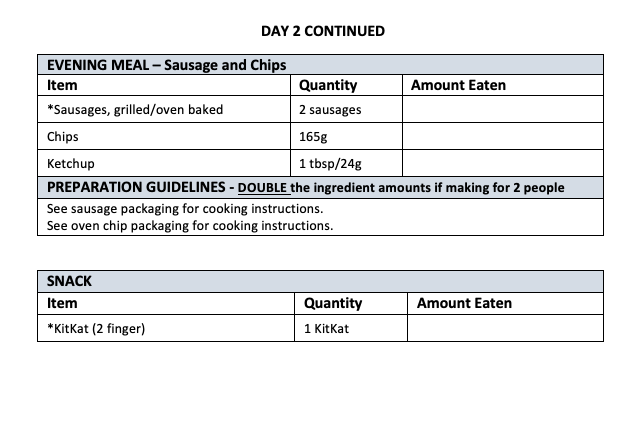

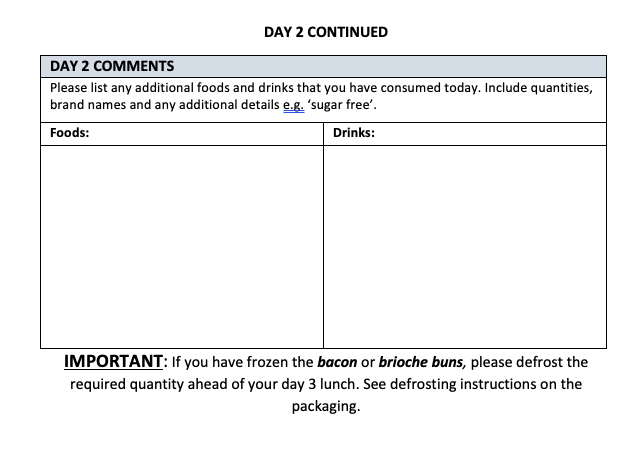

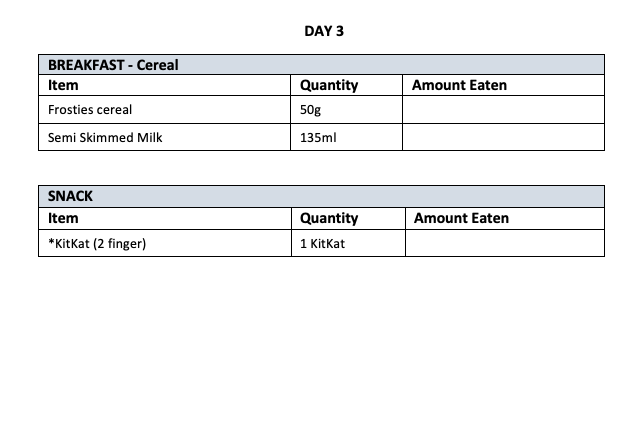

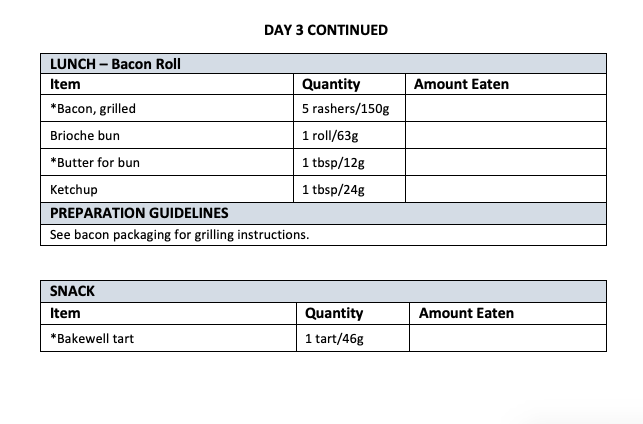

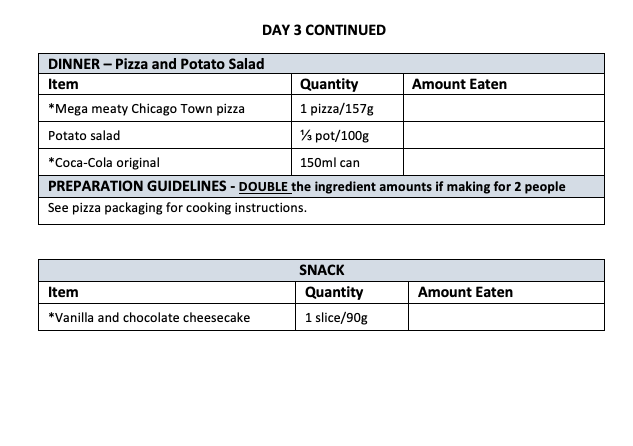

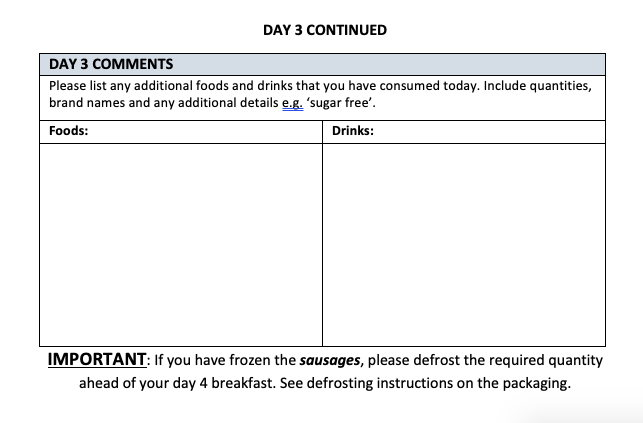

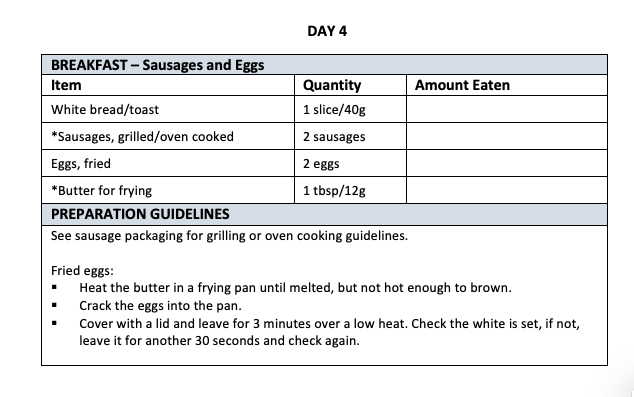

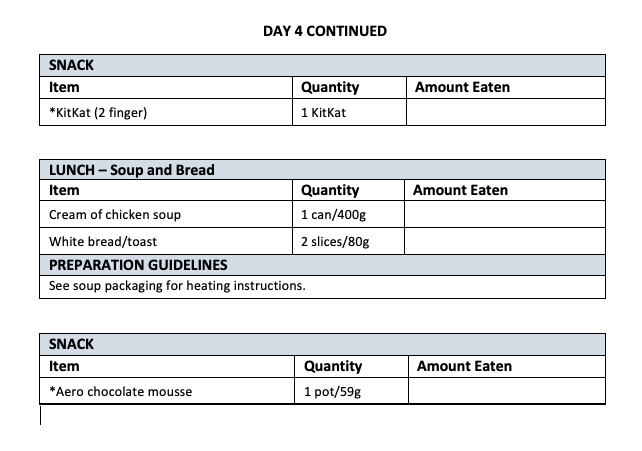

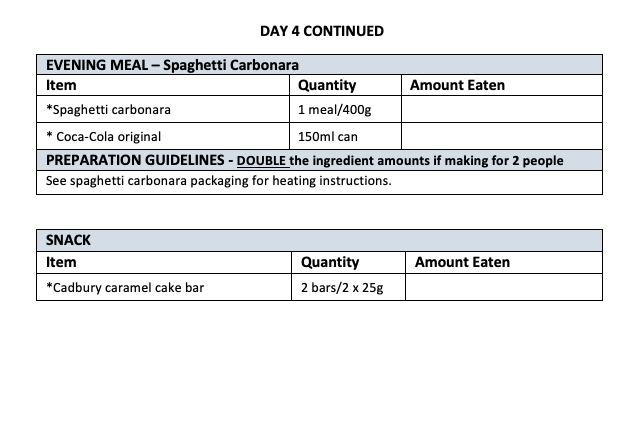

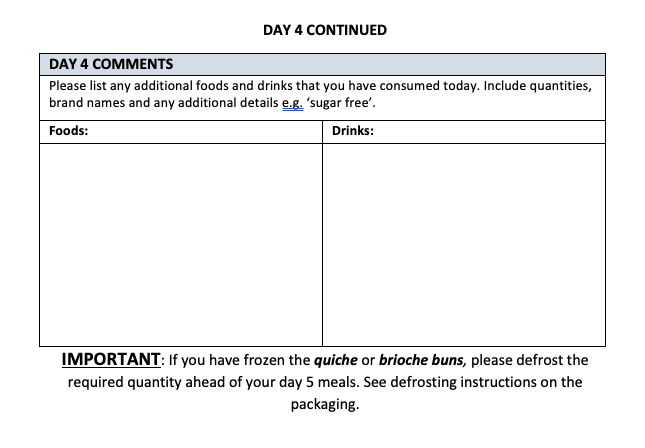

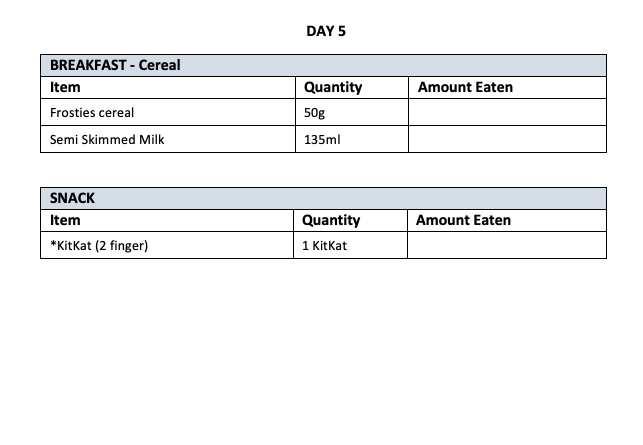

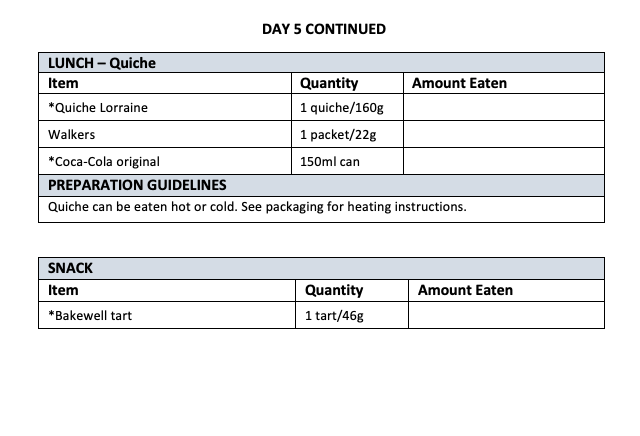

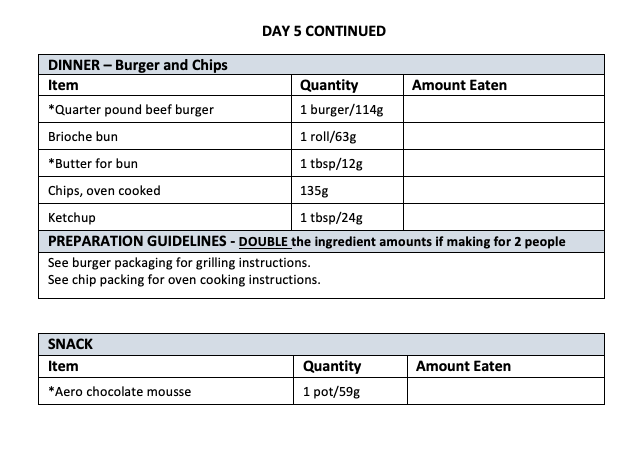

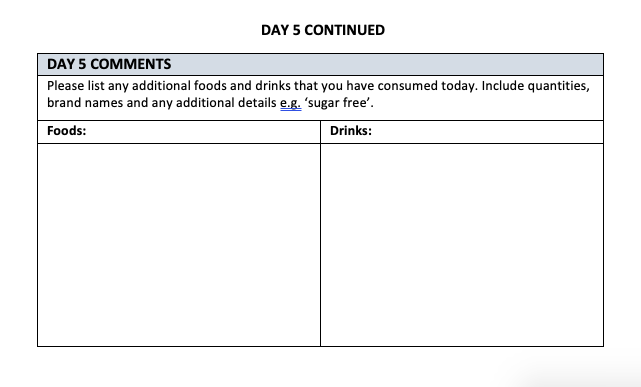


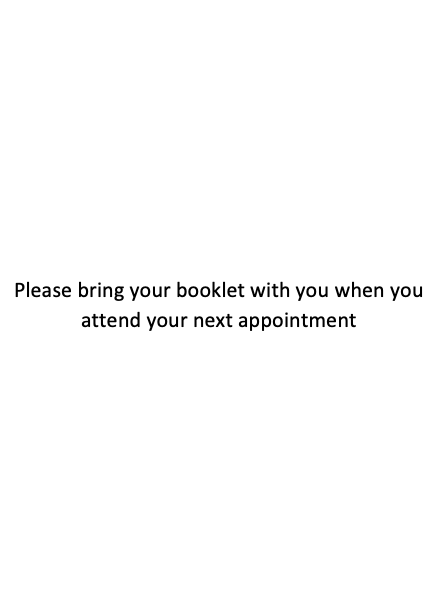

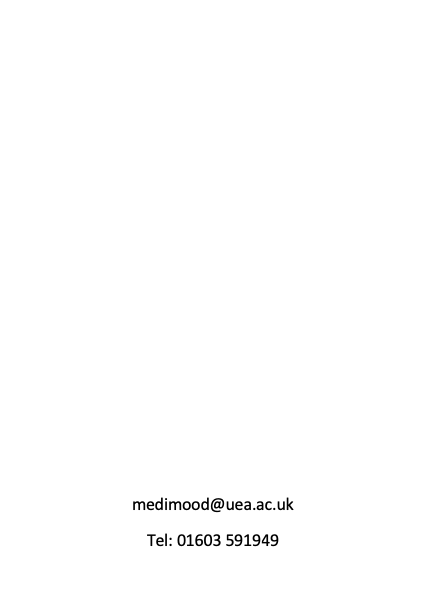

Supplement: online supplemental file 1 [file bmjopen-14-12-s001.docx]
