# Supplementary material for: Acute effect of a Mediterranean-style dietary pattern (MDP) on mood, anxiety and cognition in UK adults with mild to moderate anxiety and depression: the MediMood randomised controlled trial protocol
Source: BMJ Open. 2024 Dec 20;14(12):e082935. doi: 10.1136/bmjopen-2023-082935 (PMC11667456; doi:10.1136/bmjopen-2023-082935)
Supplement: Supplementary file 2 [file bmjopen-2023-082935.reviewer_comments.pdf]

PEER REVIEW HISTORY

BMJ Open publishes all reviews undertaken for accepted manuscripts. Reviewers are asked to complete a checklist review form and are provided with free text boxes to elaborate on their assessment. These free text comments are reproduced below.

ARTICLE DETAILS

Title (Provisional)

The acute effect of a Mediterranean-style dietary pattern (MDP) on mood, anxiety and cognition in UK adults with mild to moderate anxiety and depression: the MediMood randomised controlled trial protocol

Authors

Esgunoglu, Latife; Liaquat, Marrium; Gillings, Rachel; Lazar, Alpar; Leddy, Adrian; Brooks, Jon; Penny, William; Sami, Saber; Hornberger, M; Stevenson , Emma; Jennings, Amy; Minihane, Anne Marie

---

VERSION 1 - REVIEW

**Reviewer**	**1**
**Name**	**Ertaş Öztürk, Yasemin**
**Affiliation**	**Ondokuz Mayis Universitesi**
**Date**	**28-Dec-2023**
**COI**	**None**

Authors should add whether they enroll the participants are pregnant, breastfeeding stage of have any chronic disease?

**Reviewer**	**2**
**Name**	**Olivares, María**
**Affiliation**	**Department of Nutrition and Food Science, Faculty of Health Sciences, Melilla Campus, University of Granada**
**Date**	**25-Jan-2024**
**COI**	**None**

Thank you for the opportunity to review this manuscript entitled “The acute effect of a Mediterranean-style dietary pattern (MDP) on mood, anxiety and cognition in UK adults with mild to moderate anxiety and depression: the MediMood randomised controlled trial protocol”. (Manuscript Number: bmjopen-2023-082935)

Overall, the manuscript is of interest to the readership and the topic is of public health importance.

Comments to Author:

First of all, I would like to express my greetings for your research, in general, it is well written and very interesting.. However, below are some suggestions for researchers and readers. It is thought that resolving the deficiencies has the potential to increase the quality of the article.

Methods and analysis: In the methods section you should include the sample size calculation with an estimate of how many participants will be needed for the primary outcome to be significant. Your article includes discussion and conclusions. Please note that there is no need to include these parts in a protocol.

The rest of the sections that I have not commented on are clearly specified.

Congratulations, I hope future results are promising.

Sincerily

**Reviewer**	**3**
**Name**	**Rizvi, Zahara Ali**
**Affiliation**	**University of the Punjab Quaid-i-Azam Campus, Public Health**
**Date**	**21-May-2024**
**COI**	**Zahara Ali Rizvi, University of the Punjab**

Kindly explore more about western diet and there are any recommendations or associations with anxiety and depression, that will make your research article more worthy.

VERSION 1 - AUTHOR RESPONSE

Reviewer: 1

Authors should add whether they enrol the participants are pregnant, breastfeeding stage of have any chronic disease?

Response: *Thank you for this comment. Incident chronic disease was not a specific exclusion criterion. For MRI safety, 'any possibility of being pregnant' or those with specific medical implants or devices (such as cardiac pacemakers or artificial limbs) were precluded from participating (L159-163 and supplementary material).*

Reviewer: 2

In the methods section you should include the sample size calculation with an estimate of how many participants will be needed for the primary outcome to be significant.

Response: *The sample size calculation has been provided in L361-368*

Your article includes discussion and conclusions. Please note that there is no need to include these parts in a protocol.

Response: *Thank you. We removed the conclusion section and kept a brief discussion similar to another protocol paper our team published in the BMJ Open (https://bmjopen.bmj.com/content/11/2/e042823.abstract).*

Reviewer: 3

Kindly explore more about western diet and there are any recommendations or associations with anxiety and depression, that will make your research article more worthy.

Response: *Thank you for this comment. We have now reported on the associations between a Western diet and mental health disorders in the introduction (L102-104).*
